# Supplementary material for: Factors associated with elevated blood pressure or hypertension in Afro-Caribbean youth: a cross-sectional study
Source: PeerJ. 2018 Feb 13;6:e4385. doi: 10.7717/peerj.4385 (PMC5815333; doi:10.7717/peerj.4385)
Supplement: Supplemental Information 1 [file peerj-06-4385-s001.docx]

# **Factors associated with elevated blood pressure or hypertension in Afro-Caribbean youth: a cross-sectional study**

Trevor S Ferguson^1*^, Novie OM Younger-Coleman^1^, Marshall K Tulloch-Reid^1^, Nadia R. Bennett^1^, Amanda E. Rousseau^1^, Jennifer M Knight-Madden^1^, Maureen E Samms-Vaughan^2^, Deanna E Ashley^3^, Rainford J. Wilks^1^

**Supplementary Tables**

**Institutions: ^1^**Caribbean Institute for Health Research, The University of the West Indies, Mona, Kingston, Jamaica

^2^Department of Child Health, The University of the West Indies, Mona, Kingston, Jamaica

^3^School of Graduate Studies and Research, The University of the West Indies, Mona, Kingston, Jamaica

***Submitting Author/Author for correspondence:**

Dr. Trevor Ferguson

Epidemiology Research Unit, Caribbean Institute for Health Research, University of the West Indies, Mona, Kingston 7, Jamaica.

Telephone: (876) 927 2471 Fax: (876) 927 2984

Email: [trevor.ferguson02@uwimona.edu.jm](mailto:trevor.ferguson02@uwimona.edu.jm), [trevor.ferguson02@gmail.com](mailto:trevor.ferguson02@gmail.com)

**Table S1:** Distribution of Individual Household Possession Items

| Item | Number with item | % |
| --- | --- | --- |
| Television set | 881 | 98.2 |
| Cable television / Satellite TV | 616 | 68.8 |
| Gas / Electric Stove | 880 | 98.1 |
| Refrigerator | 850 | 94.8 |
| Freezer | 292 | 32.7 |
| Living room set | 687 | 76.6 |
| Stereo equipment | 730 | 81.5 |
| Washing machine | 506 | 56.47 |
| Cars or other vehicles | 406 | 45.5 |
| Telephone | 817 | 91.3 |
| Video Cassette Recorder | 523 | 58.5 |
| DVD / CD player | 743 | 82.8 |
| Computer | 416 | 46.3 |
| Internet | 242 | 27.1 |
| Radio / Cassette Player | 816 | 91.0 |
| Microwave oven | 628 | 70.0 |
| Air-conditioning | 67 | 7.5 |

**Table S2:** Odds ratio for elevated blood pressure or hypertension from logistic regression models assessing sex interaction for risk factors potentially associated with elevated blood pressure

|  | **Males**  **n = 409** | | **Females**  **n = 489** | | **P-value for sex interaction** |
| --- | --- | --- | --- | --- | --- |
| **Variable** | **Odds Ratio** | **95% CI** | **Odds Ratio** | **95% CI** |  |
| BMI Category |  |  |  |  |  |
| *Normal weight (18.5 -24.9 kg/m^2^)* | 1.0 | - | 1.0 | - | - |
| *Underweight (<18.5 kg/m^2^)* | 0.53 | 0.17 – 1.58 | 1.31 | 0.61 – 2.84 | 0.179 |
| *Overweight (25-29.9 kg/m^2^)* | 1.54 | 0.83 – 2.85 | 1.58 | 0.82 – 3.06 | 0.948 |
| ***Obese (≥30kg/m^2^)*** | **7.8** | **2.98 – 20.5** | **1.96** | **0.89 – 4.31** | **0.030** |
| **Central Obesity^1^** | **6.57** | **2.48 – 17.4** | **1.52** | **0.86 – 2.67** | **0.011** |
| High Glucose (upper quintile) | 2.13 | 1.34 – 3.37 | 1.18 | 0.48 – 2.95 | 0.263 |
| High triglycerides (upper quintile) | 1.80 | 1.08 – 2.98 | 1.95 | 1.09 – 3.48 | 0.839 |
| HOMA-IR (log transformed, upper quintile) | 3.48 | 1.78 – 6.75 | 1.68 | 0.92 – 3.08 | 0.115 |
| Occupation of Household Head |  |  |  |  |  |
| *Professionals/Managers* | 1.0 | - | 1.0 | - | - |
| *Office, Service, or Trade Workers* | 0.76 | 0.44 – 1.30 | 1.37 | 0.66 – 2.84 | 0.201 |
| *Semi-Skilled/Unskilled* | 0.92 | 0.50 – 1.69 | 2.15 | 0.99 – 4.71 | 0.093 |
| No. of Household Possession |  |  |  |  |  |
| *High (15-17 items)* | 1.0 | - | 1.0 | - | - |
| ***Moderate (10-14 items)*** | **0.58** | **0.33 – 1.02** | **4.32** | **1.30 – 14.4** | **0.003** |
| *Low (0-9 items)* | 0.80 | 0.43 – 1.51 | 2.74 | 0.78 – 9.65 | 0.088 |
| Alcohol Consumption |  |  |  |  |  |
| *Never Drank Alcohol* | 1.0 | - | 1.0 | - | - |
| *Rarely Drinks Alcohol* | 1.01 | 0.38 – 2.65 | 0.60 | 0.29 – 1.23 | 0.392 |
| *Drinks 1-2 times/week* | 1.34 | 0.52 – 3.46 | 0.58 | 0.25 – 1.32 | 0.190 |
| *Drinks ≥3 times/week* | 1.16 | 0.45 – 2.94 | 0.46 | 0.19 – 1.12 | 0.164 |

^1^Central obesity defined as waist circumference ≥ 94 cm in males and ≥80 cm in females

Estimates derived from separate models for each risk factor with test for interaction between sex and the given risk factor.

**Table S3:** Number of missing values and number of observations for variables used in the analysis.

| Variables | Males | | Females | |
| --- | --- | --- | --- | --- |
|  | Number Missing | Number of Observations | Number Missing values | Number of Observations |
| Waist circumference | 0 | 409 | 1 | 488 |
| Waist to hip Ratio | 0 | 409 | 1 | 488 |
| White blood cell count | 12 | 397 | 17 | 472 |
| Fasting Glucose | 17 | 392 | 16 | 473 |
| Total cholesterol | 19 | 390 | 13 | 476 |
| HDL Cholesterol | 28 | 381 | 22 | 467 |
| LDL cholesterol | 32 | 377 | 23 | 466 |
| Triglycerides | 19 | 390 | 13 | 476 |
| Creatinine | 21 | 388 | 24 | 465 |
| Urine albumin | 17 | 392 | 21 | 468 |
| High sensitivity CRP | 41 | 368 | 66 | 423 |
| Glomerular filtration rate | 21 | 388 | 25 | 464 |
| Fasting Insulin | 57 | 352 | 58 | 431 |
| Family History of HTN | 86 | 323 | 83 | 406 |
| Chronic Kidney Disease | 29 | 380 | 35 | 454 |
| Albuminuria | 17 | 392 | 21 | 468 |
| Parental Education | 69 | 340 | 50 | 439 |
| Parental Occupation | 34 | 375 | 27 | 462 |
| Household Possessions | 1 | 408 | 0 | 489 |
| Physical Activity Level | 1 | 408 | 0 | 489 |
| Current Smoking | 1 | 408 | 0 | 489 |
| Current Marijuana use | 3 | 406 | 1 | 488 |
| Alcohol use | 4 | 405 | 4 | 485 |

**Table S4: Summary values for observed and imputed data used in the analyses**

| Characteristic | Observed |  | Imputed |  |
| --- | --- | --- | --- | --- |
|  | N^1^ | Mean / Percentage^2^ | N^1^ | Mean / Percentage^2^ |
| White blood cell count (cells X 10^9^/L) | 869 | 5.87 (1.87) | 29 | 5.97 (1.92) |
| Creatinine (mmol/L) | 853 | 67.7 (24.7) | 45 | 66.5 (24.8) |
| Elevated waist circumference^3^ | 897 | 15.6% | 1 | 20.0% |
| High glucose (upper quintile) | 865 | 17.6% | 33 | 15.6% |
| High cholesterol (≥5.2 mmol/l) | 877 | 14.4% | 21 | 12.0% |
| Low HDL^4^ | 848 | 46.1% | 50 | 40.0% |
| High triglycerides (upper quintile) | 866 | 20.6% | 32 | 18.9% |
| High HOMA-IR^5^ (upper quintile) | 750 | 19.5% | 148 | 20.5% |
| Albuminuria^6^ | 860 | 6.9% | 38 | 9.1% |
| High hsCRP^7^ (>3 mg/L) | 791 | 14.4% | 107 | 21.4% |
| Family history of hypertension | 729 | 21.4% | 169 | 45.1% |
| Current Cigarette Smoking | 897 | 9.6% | 1 | 28.0% |
| Current Marijuana Smoking | 894 | 18.1% | 4 | 45.0% |
| Highest Education of Parent/Guardian | 779 |  | 119 |  |
| *Post-Secondary* |  | 28.2% |  | 19.2% |
| *Secondary* |  | 58.2% |  | 59.2% |
| *Less than Secondary* |  | 13.6% |  | 21.6% |
| Occupation of Household Head | 837 |  | 61 |  |
| *Professionals/Managers* |  | 24.1% |  | 21.6% |
| *Office, Service or Trade Workers* |  | 50.2% |  | 52.5% |
| *Semi-Skilled/Unskilled Workers* |  | 25.7% |  | 25.9% |
| Number of Household Possession | 897 |  | 1 |  |
| *High (15-17 items)* |  | 15.2 |  | 8.0 |
| *Moderate (10-14 items)* |  | 55.4 |  | 52.0 |
| *Low (0-9 items)* |  | 29.4 |  | 40.0 |
| Physical Activity Level | 897 |  | 1 |  |
| *High* |  | 24.2 |  | 52.0 |
| *Moderate* |  | 41.7 |  | 32.0 |
| *Low* |  | 34.1 |  | 16.0 |
| Alcohol Consumption | 890 |  | 8 |  |
| *Never Drank Alcohol* |  | 10.1 |  | 11.5 |
| *Rarely Drinks Alcohol* |  | 36.6 |  | 33.0 |
| *Drinks Alcohol 1-2 times/week* |  | 25.4 |  | 20.0 |
| *Drinks Alcohol ≥3 times/week* |  | 29.9 |  | 35.5 |
|  |  |  |  |  |

^1^N represents number of observed values and number of imputed values for each of 25 imputed datasets

^2^Summary statistics are mean and standard deviations for continuous variables (white blood cell count and creatinine and percentage with characteristic for categorical variables. Values for imputed data represent the pooled values over 25 imputations

^3^Elevated waist circumference defined as ≥94 cm for males and ≥80 cm for females

^4^HDL = High density lipoprotein cholesterol; defined as <1.0 mmol/l for males and <1.3 mmol/l for females

^5^HOMA-IR = Homeostasis model assessment insulin resistance

^6^Albuminuria = albumin to creatinine ratio ≥30 mg/g

^7^hsCRP = high sensitivity C-reactive protein

**Table S5:** Complete Case Analysis: Factors Associated with Elevated Blood Pressure or hypertension (BP ≥120/80) in Multivariable Logistic Regression Models among Male and Female Young Adults in the Jamaica 1986 Birth Cohort

|  | **Males** (n=306) | | | **Females** (n=409) | | |
| --- | --- | --- | --- | --- | --- | --- |
| **Variable** | **Odds Ratio** | **95% CI** | **P-Value** | **Odds Ratio** | **95% CI** | **P-Value** |
| Age (years) | 1.69 | 1.05 - 2.71 | 0.030 | 2.67 | 1.62 – 4.40 | <0.001 |
| Height (cm) | - | - | - | 1.08 | 1.03 - 1.13 | 0.003 |
| BMI Category |  |  |  |  |  |  |
| *Normal weight (18.5 -24.9 kg/m^2^)* | 1.0 | - | - | 1.0 | - | - |
| *Underweight (<18.5 kg/m^2^)* | 0.47 | 0.13 – 1.71 | 0.250 | 2.00 | 0.84 – 4.73 | 0.117 |
| *Overweight (25-29.9 kg/m^2^)* | 1.61 | 0.76 – 3.41 | 0.214 | 1.43 | 0.65 – 3.16 | 0.371 |
| *Obese (≥30kg/m^2^)* | 9.58 | 2.26 – 40.5 | 0.002 | 1.65 | 0.62 – 4.41 | 0.3.17 |
| High Glucose (upper quintile) | 1.94 | 1.08 – 3.48 | 0.027 | - | - | - |
| High Triglycerides (upper quintile) | - | - | - | 1.82 | 0.88– 3.73 | 0.104 |
| HOMA-IR (log transformed, upper quintile) | 2.08 | 0.91 – 4.78 | 0.083 | 1.95 | 0.96 – 3.93 | 0.063 |
| High hsCRP | 0.31 | 0.10 – 0.93 | 0.037 | - | - | - |
| White blood cell count | - | - |  | 1.10 | 0.95 – 1.29 | 0.209 |
| Household possessions |  |  |  |  |  |  |
| *High (15-17 items)* | 1.0 | - | - | 1.0 | - | - |
| *Moderate (10-14 items)* | 0.63 | 0.30 – 1.34 | 0.232 | 3.64 | 1.01 – 13.1 | 0.047 |
| *Low (0-9 items)* | 1.32 | 0.58 – 3.02 | 0.509 | 2.31 | 0.61 – 8.83 | 0.220 |
| Physical Activity Level |  |  |  |  |  |  |
| *High Physical Activity Level* | 1.0 | - | - | 1.0 | - | - |
| *Moderate Physical Activity Level* | 0.50 | 0.28 – 0.91 | 0.026 | 0.88 | 0.35 – 2.26 | 0.798 |
| *Low Physical Activity Level* | 0.44 | 0.20 – 0.96 | 0.040 | 0.50 | 0.19 – 1.31 | 0.159 |
| Alcohol Consumption |  |  |  |  |  |  |
| *Never Drank Alcohol* | - | - | - | 1.0 | - | - |
| *Rarely Drinks Alcohol (<once/week)* | - | - | - | 0.47 | 0.19 – 1.13 | 0.091 |
| *Drinks Alcohol 1-2 times/week* | - | - | - | 0.50 | 0.18– 1.36 | 0.174 |
| *Drinks Alcohol ≥3 times/week* |  | - | - | 0.43 | 0.15 – 1.22 | 0.113 |

BMI = Body mass Index; HOMA-IR = Homeostasis Model Assessment Insulin Resistance; hsCRP = high sensitivity C-reactive protein.

Separate models created for males and females. Models for males included age, BMI category, high glucose, high HOMA-IR, High hsCRP, possession category and physical activity levels. Models for females included age, height BMI category, high triglycerides, high HOMA-IR, possession category. physical activity levels and alcohol consumption categories.

**Table S6**: Summary statistics for participant characteristics and factors putatively associated with hypertension for males and females stratified by blood pressure category

| Characteristic | Males | | | Females | | |
| --- | --- | --- | --- | --- | --- | --- |
|  | Normal BP  Mean ± SD  n = 287 | EBP/HTN  Mean ± SD  n = 122 | P-value | Normal BP  Mean ± SD  n =423 | EBP/HTN  Mean ± SD  n = 66 | P-value |
| Age (years) | 18.8 ± 0.60 | 18.9 ± 0.56 | 0.029 | 18.7 ± 0.62 | 19.0 ± 0.59 | <0.001 |
| Weight (kg) | 68.5 ± 10.8 | 77.2 ± 18.8 | <0.001 | 61.8 ± 15.1 | 65.9 ± 17.8 | 0.079 |
| Height (cm) | 176.6 ± 6.2 | 177.3 ± 7.0 | 0.330 | 163.3 ± 6.0 | 165.4 ± 6.4 | 0.012 |
| Body mass index (kg/m^2^) | 22.0 ± 3.1 | 24.5 ± 5.9 | <0.001 | 23.2 ± 5.5 | 24.0 ± 6.2 | 0.295 |
| Systolic BP (mmHg) | 109.0 ± 6.9 | 125.2 ± 8.3 | <0.001 | 105.5 ± 7.3 | 119.0 ± 8.8 | <0.001 |
| Diastolic BP (mmHg) | 66.6 ± 8.6 | 75.5 ± 11.5 | <0.001 | 65.3 ± 8.0 | 77.5 ± 9.4 | <0.001 |
| Waist circumference (cm) | 73.2 ± 6.9 | 79.8 ± 15.9 | <0.001 | 73.4 ± 11.7 | 76.6 ± 14.0 | 0.085 |
| Hip circumference (cm) | 92.8 ± 6.9 | 98.0 ± 11.7 | <0.001 | 96.0 ± 10.8 | 98.8 ± 12.0 | 0.078 |
| Waist-to-Hip ratio | 0.79 ± 0.04 | 0.81 ± 0.13 | 0.031 | 0.77 ± 0.15 | 0.77 ± 0.06 | 0.659 |
| White blood cells (cells X 10^9^/L) | 5.3 ± 1.6 | 5.3 ± 1.4 | 0.848 | 6.3 ± 2.0 | 6.8 ± 1.9 | 0.079 |
| Glucose (mmol/L) | 4.7 ± 0.5 | 4.9 ± 0.7 | 0.005 | 4.4 ± 0.4 | 4.5 ± 0.4 | 0.102 |
| Total cholesterol (mmol/L) | 4.1 ± 0.8 | 4.3 ± 0.8 | 0.001 | 4.5 ± 0.9 | 4.6 ± 1.1 | 0.312 |
| HDL cholesterol (mmol/L) | 1.1 ± 0.2 | 1.1 ± 0.2 | 0.807 | 1.3 ± 0.3 | 1.2 ± 0.3 | 0.438 |
| LDL cholesterol (mmol/L) | 2.7 ± 0.7 | 2.9 ± 0.7 | 0.003 | 3.0 ± 0.8 | 3.1 ± 1.0 | 0.410 |
| Triglycerides (mmol/L) | 0.58 ± 0.23 | 0.66 ± 0.33 | 0.014 | 0.55 ± 0.24 | 0.65 ± 0.33 | 0.028 |
| Creatinine (μmol/L) | 81.0 ± 15.9 | 79.3 ± 16.4 | 0.343 | 56.8 ± 27.1 | 57.3 ± 12.3 | 0.805 |
|  | Median (p25, p75) | | | Median (p25, p75) | | |
| Urinary albumin (mg/g) | 4 (3, 7) | 4 (3, 8) | 0.838 | 5 (3, 11) | 4.9 (3, 10) | 0.923 |
| hsCRP (mg/L) | 0.5 (0.3, 1.3) | 0.5 (0.3, 1.3) | 0.675 | 0.9 (0.3, 2.2) | 1.3 (0.5, 2.9) | 0.061 |
| Fasting Insulin (pmol/L) | 4.0 (2.5, 6.3) | 5.9 (3.3, 8.6) | 0.001 | 6.8 (4.2, 10) | 6.8 (3.4, 12.2) | 0.847 |
| HOMA-IR | 0.5 (0.3, 0.8) | 0.7 (0.4, 1.1) | 0.005 | 0.9 (0.5, 1.2) | 0.9 (0.5, 1.5) | 0.914 |

EBP/HTN = elevated blood pressure or hypertension; HDL = high density lipoprotein; LDL = low density lipoprotein; eGFR = estimated glomerular filtration rate. IQR = interquartile range

There were missing values for some participants (maximum n= 66 for hsCRP among women. Full list of number of missing values for each variable is shown in Table S4

HDL = high density lipoprotein; LDL = low density lipoprotein; hsCRP = high sensitivity C-reactive protein.

**Table S7:** Proportion of participants with CVD risk factors by blood pressure category for males and females stratified by blood pressure category

| Characteristic | Males | | | Females | | |
| --- | --- | --- | --- | --- | --- | --- |
|  | Normal BP  % (n)  n = 287 | EBP/HTN  % (n)  n = 122 | P-value | Normal BP  % (n)  n =423 | EBP/HTN  % (n)  n = 66 | P-value |
| Body Mass Index categories |  |  |  |  |  |  |
| *Underweight (<18.5 kg/m^2^)* | 7.3 (21) | 3.3 (4) | <0.001 | 14.2 (61) | 15.2 (10) | 0.294 |
| *Normal weight (18.5 -24.9 kg/m^2^)* | 78.8 (226) | 67.2 (82) |  | 56.7 (240) | 45.5 (30) |  |
| *Overweight (25-29.9 kg/m^2^)* | 11.8 (34) | 15.6 (19) |  | 19.2 (81) | 24.2 (16 |  |
| *Obese (≥30kg/m^2^)* | 2.1 (6) | 13.9 (17) |  | 9.7 (41) | 15.2 (10) |  |
| Central obesity^1^ | 2.1 (6) | 12.3 (15) | <0.001 | 23.2 (98) | 31.8 (21) | 0.130 |
| High waist-to-hip ratio^2^ | 0 (0) | 3.3 (4) | 0.008 | 19.9 (84) | 22.7 (15) | 0.596 |
| Albuminuria | 4.8 (13) | 5.9 (7) | 0.643 | 8.2 (33) | 9.2 (6) | 0.778 |
| Highest Education of Parent/Guardian^3^ |  |  |  |  |  |  |
| *Post-Secondary* | 25.4 (62) | 28.1 (27) | 0.781 | 30.7 (117) | 24.1 (14) | 0.591 |
| *Secondary* | 61.9 (151) | 61.5 (59) |  | 54.6 (208) | 60.3 (35) |  |
| *Less than Secondary* | 12.7 (31) | 10.4 (10) |  | 14.7 (56) | 15.5 (9) |  |
| Occupation of Household Head |  |  |  |  |  |  |
| *Professionals/Managers* | 22.3 (58) | 26.1 (30) | 0.551 | 25.8 (104) | 17.7 (11) | 0.130 |
| *Office, Service or Trade Workers* | 51.2 (133) | 45.2 (52) |  | 51.3 (205) | 48.4 (30) |  |
| *Semi-Skilled/Unskilled Workers* | 26.5 (69) | 28.7 (33) |  | 23.0 (92) | 33.9 (21) |  |
| Number of Household Possession |  |  |  |  |  |  |
| *High (15-17 items)* | 15.0 (43) | 21.5 (26) | 0.122 | 15.0 (64) | 4.6 (3) | 0.019 |
| *Moderate (10-14 items)* | 59.9 (172) | 49.6 (60) |  | 52.0 (220) | 68.2 (45) |  |
| *Low (0-9 items)* | 25.1 (72) | 28.9 (35) |  | 32.9 (139) | 27.3 918) |  |
| Physical Activity Level |  |  |  |  |  |  |
| *High* | 35.9 (103) | 41.3 (50) | 0.520 | 12.5 (53) | 16.7 (11) | 0.449 |
| *Moderate* | 45.0 (129) | 43.0 (52) |  | 39.0 (165) | 42.4 (28) |  |
| *Low* | 19.2 (55) | 15.7 (19) |  | 48.5 (205) | 40.9 (27) |  |
| Current Cigarette Smoking | 13.6 (39) | 14.1 (17) | 0.902 | 6.2 (26) | 6.1 (4) | 0.978 |
| Current Marijuana Smoking | 31.2 (89) | 31.4 (38) | 0.972 | 7.3 (31) | 6.2 (4) | 0.733 |
| Alcohol Consumption |  |  |  |  |  |  |
| *Never Drank Alcohol* | 6.7 (19) | 5.8 (7) | 0.785 | 12.2 (51) | 19.7 (13) | 0.360 |
| *Rarely Drinks Alcohol* | 27.5 (78) | 24.0 (29) |  | 45.4 (190) | 43.9 (29) |  |
| *Drinks Alcohol 1-2 times/week* | 27.8 (79) | 32.2 (39) |  | 22.4 (94) | 21.2 (14) |  |
| *Drinks Alcohol ≥3 times/week* | 38.0 (108) | 38.0 (46) |  | 20.1 (84) | 15.2 (10) |  |

EBP/HTN = elevated blood pressure or hypertension;

^1^Central obesity defined as waist circumference ≥ 94 cm in males and ≥80 cm in females

^2^High waist-to-hip ratio ≥0.95 for males and ≥0.80 for females

^3^Education category “post-secondary” includes persons with vocational training, college, or university education; secondary corresponds to high school (up to grade 11); less than secondary corresponds to persons who had only elementary school education or persons who did not complete high school (i.e. high school grade 10 or below)

**Table S8A:** Regression Coefficients for variables in Multivariable ANOVA Models of Systolic Blood Pressure among Male and Female Young Adults in the Jamaica 1986 Birth Cohort

|  | **Males**  (n=409) | | | **Females**  (n=489) | | | |  |
| --- | --- | --- | --- | --- | --- | --- | --- | --- |
| **Variable** | **β coefficient** | **95% CI** | **P-Value** | | **β coefficient** | **95% CI** | **P-Value** | |
| Age (years) | 1.96 | 0.30, 3.62 | 0.021 | | 1.99 | 0.75, 3.24 | 0.002 | |
| Height (cm) | - | - | - | | 0.14 | 0.02, 0.26 | 0.026 | |
| BMI Category |  |  |  | |  |  |  | |
| *Normal weight (18.5 -24.9 kg/m^2^)* | 0 | - | - | | - |  |  | |
| *Underweight (<18.5 kg/m^2^)* | -1.28 | -5.28, 2.71 | 0.529 | | -1.11 | -3.33, 1.11 | 0.326 | |
| *Overweight (25-29.9 kg/m^2^)* | 4.03 | 1.14, 6.91 | 0.006 | | 0.86 | -1.14, 2.87 | 0.398 | |
| *Obese (≥30kg/m^2^)* | 9.76 | 5.01, 14.5 | <0.001 | | 5.19 | 2.48, 7.91 | <0.001 | |
| High Glucose (upper quintile) | 4.81 | 2.61, 7.00 | <0.001 | | 2.32 | -0.54, 5.18 | 0.112 | |
| High Cholesterol (≥5.2 mmol/l) |  |  |  | | 1.57 | -0.37, 3.50 | 0.113 | |
| White blood cell count | - | - | - | | 0.31 | -0.08, 0.70 | 0.122 | |
| High hsCRP | -2.20 | -5.70, 1.30 | 0.218 | | - | - | - | |
| HOMA-IR (log transformed, upper quintile) | 2.68 | -0.81, 6.18 | 0.131 | | 1.66 | -0.38, 3.71 | 0.110 | |
| Physical Activity Level |  |  |  | |  |  |  | |
| *High* | - |  |  | | - | - | - | |
| *Moderate* | -0.41 | -2.59, 1.77 | 0.713 | | - | - | - | |
| *Low* | -2.41 | -2.56, 4.03 | 0.088 | | - | - | - | |
| Parental Occupation |  |  |  | |  |  |  | |
| *Professionals/Managers* | - |  |  | | - |  |  | |
| *Office, Service or Trade* | 0.37 | -2.05, 2.80 | 0.761 | | 1.11 | -0.79, 3.01 | 0.250 | |
| *Semi-Skilled/Unskilled* | 2.50 | -0.23, 5.24 | 0. 073 | | 2.26 | 0.04, 4.49 | 0.046 | |
| Alcohol Consumption |  |  |  | |  |  |  | |
| *Never Drank Alcohol* | - |  |  | | - |  |  | |
| *Rarely Drinks Alcohol* | - | - | - | | -2.41 | -4.78, -0.05 | 0.045 | |
| *Drinks Alcohol 1-2 times/week* | - | - | - | | -1.89 | -4.53, 0.74 | 0.158 | |
| *Drinks Alcohol ≥3 times/week* | - | - | - | | -2.58 | -5.29, 0.12 | 0.061 | |
| Current Cigarette Smoking | 3.00 | 0.24, 5.76 | 0.033 | | - | - | - | |

Separate multiple imputation models created for males and females. Each sex-specific model included variables as shown in the table.

**Table S8B:** Regression Coefficients for variables in Multivariable ANOVA Models of Diastolic Blood Pressure among Male and Female Young Adults in the Jamaica 1986 Birth Cohort

|  | **Males** (n=409) | | | **Females** (n=489) | | |
| --- | --- | --- | --- | --- | --- | --- |
| **Variable** | **β coefficient** | **95% CI** | **P-Value** | **β coefficient** | **95% CI** | **P-Value** |
| Age (years) | 4.94 | 3.28, 6.60 | <0.001 | 3.08 | 1.81, 4.37 | <0.001 |
| Height (cm) | - | - | - | 0.16 | 0.03, 0.29 | 0.015 |
| BMI Category |  |  |  |  |  |  |
| *Normal weight (18.5 -24.9 kg/m^2^)* | - |  |  | - |  |  |
| *Underweight (<18.5 kg/m^2^)* |  |  |  | 0.94 | -1.38, 3.27 | 0.426 |
| *Overweight (25-29.9 kg/m^2^)* |  |  |  | 1.88 | -0.19, 3.94 | 0.075 |
| *Obese (≥30kg/m^2^)* |  |  |  | 2.11 | -0.56, 4.79 | 0.122 |
| High Glucose (upper quintile) | 2.20 | -0.02, 4.43 | 0.052 | - | - | - |
| High Triglycerides (upper quintile) | - | - |  | -2.01 | -4.01, -0.02 | 0.048 |
| High HOMA-IR (log transformed, upper quintile) | 2.44 | -0.78, 5.66 | 0.137 | - | - | - |
| Low HDL | 2.24 | -0.03, 4.52 | 0.054 | - | - | - |
| Family history of hypertension | 1.89 | -0.23, 4.00 | 0.080 | - | - | - |
| White blood cell count | - | - | - | -0.28 | -0.70, 0.13 | 0.177 |
| Household possessions |  |  |  |  |  |  |
| *High (15-17 items)* | - |  |  | - |  |  |
| *Moderate (10-14 items)* | 0.87 | -1.83, 3.58 | 0.528 | - | - | - |
| *Low (0-9 items)* | 2.70 | -0.37, 5.77 | 0.085 | - | - | - |
| Physical Activity Level |  |  |  |  |  |  |
| *High* | - |  |  | - |  |  |
| *Moderate* | -0.89 | -3.04, 1.26 | 0.416 | -1.32 | -3.85, 1.21 | 0.306 |
| *Low* | -2.29 | -5.05, 0.47 | 0.104 | 2.56 | -5.05, -0.06 | 0.044 |
| Alcohol Consumption |  |  |  |  |  |  |
| *Never Drank Alcohol* | - |  |  | - |  |  |
| *Rarely Drinks Alcohol* | 5.11 | 0.92, 9.30 | 0.017 | -3.42 | -5.90, -0.95 | 0.007 |
| *Drinks Alcohol 1-2 times/week* | 5.92 | 1.76, 10.1 | 0.005 | -1.24 | -4.00, 1.51 | 0.377 |
| *Drinks Alcohol ≥3 times/week* | 6.49 | 2.32, 10.7 | 0.002 | -4.35 | -7.18, -1.53 | 0.003 |
| Currently smokes marijuana | -3.31 | -5.59, -1.03 | 0.004 | - | - | - |

Separate multiple imputation models created for males and females. Each sex-specific model included variables as shown in the table.

**Table S9:** Factors Associated with hypertension (BP ≥130/80) in Multivariable Logistic Regression Models among Male and Female Young Adults in the Jamaica 1986 Birth Cohort

|  | **Males** (n=409) | | | **Females** (n=489) | | |
| --- | --- | --- | --- | --- | --- | --- |
| **Variable** | **Odds Ratio** | **95% CI** | **P-Value** | **Odds Ratio** | **95% CI** | **P-Value** |
| Age (years) | 2.41 | 1.47- 3.97 | 0.001 | 2.57 | 1.48 – 4.47 | 0.001 |
| Height (cm) | - | - | - | 1.06 | 1.00 - 1.12 | 0.035 |
| Central obesity | 2.56 | 0.85 – 7.71 | 0.095 | - | - | - |
| High Glucose (upper quintile) | 2.30 | 1.26 – 4.18 | 0.007 | - | - | - |
| High Triglycerides (upper quintile) | - | - | - | 2.56 | 1.23 – 5.31 | 0.012 |
| HOMA-IR (log transformed, upper quintile) | 2.10 | 0.88 – 5.01 | 0.094 | - | - | - |
| Household possessions |  |  |  |  |  |  |
| *High (15-17 items)* | 1.0 | - | - | 1.0 | - | - |
| *Moderate (10-14 items)* | 0.58 | 0.28 – 1.20 | 0.143 | 7.04 | 0.93 – 53.4 | 0.059 |
| *Low (0-9 items)* | 0.78 | 0.34 – 1.81 | 0.566 | 3.52 | 0.43 – 28.7 | 0.240 |
| Physical Activity Level |  |  |  |  |  |  |
| *High Physical Activity Level* | 1.0 | - | - | - | - | - |
| *Moderate Physical Activity Level* | 0.63 | 0.34– 1.17 | 0.144 | - | - | - |
| *Low Physical Activity Level* | 0.35 | 0.14 – 0.89 | 0.027 | - | - | - |

BMI = Body mass Index; HOMA-IR = Homeostasis Model Assessment Insulin Resistance; hsCRP = high sensitivity C-reactive protein.

Separate multiple imputation models created for males and females. Models for males included age, BMI category, high glucose, high HOMA-IR, High hsCRP, possession category and physical activity levels. Models for females included age, height BMI category, high triglycerides, high HOMA-IR, possession category. physical activity levels and alcohol consumption categories.
