# Supplementary material for: Factors associated with elevated blood pressure or hypertension in Afro-Caribbean youth: a cross-sectional study
Source: PeerJ. 2018 Feb 13;6:e4385. doi: 10.7717/peerj.4385 (PMC5815333; doi:10.7717/peerj.4385)
Supplement: Supplemental Information 2 [file peerj-06-4385-s002.pdf]

Confidential Interviewer's ID # □□□□

# Impact of Early Life Experiences on Cardio-Respiratory Risk and Bone Mineral Density in Jamaican Adolescents

*Good morning. Thank you for participating in the study. I will be asking you some questions. Please take your time to answer each question as accurately as possible*

## SECTION 1: DEMOGRAPHY/EDUCATION/RELIGION

1.1 ID NUMBER: □□□□□□□□ ***(use same ID number as in previous study)***

1.1a NEW ID #: □□□□□□

1.2 Date of Interview □□□□/□□/□□ (year/month/day)

1.3 SURNAME: □□□□□□□□□□□□□□□□□□

1.4 FIRST NAME: □□□□□□□□□□□□

1.5 MIDDLE NAME: □□□□□□□□□□□□

1.6 MARITAL STATUS: 1□SINGLE 2□MARRIED 3□ DIVORCED

1.7 MOTHER'S NAME: ____________________________________

1.8 FATHER'S NAME: ____________________________________

1.9 AGE OF PARENTS: a. □□MOTHER b. □□FATHER c. □ N/R

d. □ **DK**

1.10 GENDER: 0□ Male 1□ Female

1.11 Date of Birth □□□□/□□/□□ (YEAR/MONTH/DAY)

1.12 Age at last birth day □□years

1.13 HOME ADDRESS:_____________________________________________

_____________________________________________________________

_____________________________________________________________

## SECTION 1 cont'd: DEMOGRAPHY/EDUCATION/RELIGION

***(D/K = I don't know, N/A = Not applicable, N/R = No Response)***

1.14 Are you currently: 1☐In School 2☐In College/University
3☐ Working 4☐At Home/ Unemployed 99☐N/R

1.15 Name of School or College or Workplace:___________________________________
8☐N/A 99☐N/R

1.16 What is your highest level of education completed?
0☐None 1☐All Age/ Primary 2☐ Secondary/Technical/High School
3☐College / University 9☐D/K 99☐N/R

1.17 Are you currently employed? 0☐ No 1☐Yes 99☐N/R

If yes, give type of employment: (*If no, go to question 1.18)*
1☐ Full time (≥ 30hours /week) 2☐ Part time (≤ 29 hour/ week)
3☐Self employed

1.18 What is your religious affiliation?
0☐None 1☐Christian 2☐Rastafarian 3☐Muslim 4☐Jewish
5☐ other, specify ____________________ 8☐D/K 9☐N/R

1.19 If Christian, What denomination?
1☐ Roman Catholic 2☐United/Baptist/Anglican/ Methodist
3☐ Church of God/ Pentecostal 4☐Seventh Day Adventist
5☐ Other______________ 8☐N/A 99☐N/R

1.20 Are you actively practicing your religion?
0☐No 1☐yes 8☐N/A 9☐D/ K 99☐ No Response

# SECTION 2: FAMILY MEDICAL HISTORY

*Have any of the following members of your family ever had the illnesses listed below?*

*(Please place a (√) tick in the appropriate box to indicate your response)*

(**D/K = I don't know, N/A = Not applicable, N/R = No Response**)

**2.1 High blood pressure**

| | | | | | | |
|---|---|---|---|---|---|---|
| a. | Mother | 0☐No | 1☐Yes | 8☐N/A | 9☐D/K | 99☐N/R |
| b. | Father | 0☐No | 1☐Yes | 8☐N/A | 9☐D/K | 99☐N/R |
| c. | Sister | 0☐No | 1☐Yes | 8☐N/A | 9☐D/K | 99☐N/R |
| d. | Brother | 0☐No | 1☐Yes | 8☐N/A | 9☐D/K | 99☐N/R |
| e. | Grandparent | 0☐No | 1☐Yes | 8☐N/A | 9☐D/K | 99☐N/R |
| f. | Other | 0☐No | 1☐Yes | 8☐N/A | 9☐D/K | 99☐N/R |

If other, specify ________________________________________________

**2.2 Diabetes mellitus (sugar)**

| | | | | | | |
|---|---|---|---|---|---|---|
| a. | Mother | 0☐No | 1☐Yes | 8☐N/A | 9☐D/K | 99☐N/R |
| b. | Father | 0☐No | 1☐Yes | 8☐N/A | 9☐D/K | 99☐N/R |
| c. | Sister | 0☐No | 1☐Yes | 8☐N/A | 9☐D/K | 99☐N/R |
| d. | Brother | 0☐No | 1☐Yes | 8☐N/A | 9☐D/K | 99☐N/R |
| e. | Grandparent | 0☐No | 1☐Yes | 8☐N/A | 9☐D/K | 99☐N/R |
| f. | Other | 0☐No | 1☐Yes | 8☐N/A | 9☐D/K | 99☐N/R |

If other, specify ________________________________________________

**2.3 Heart attack**

| | | | | | | |
|---|---|---|---|---|---|---|
| a. | Mother | 0☐No | 1☐Yes | 8☐N/A | 9☐D/K | 99☐N/R |
| b. | Father | 0☐No | 1☐Yes | 8☐N/A | 9☐D/K | 99☐N/R |
| c. | Sister | 0☐No | 1☐Yes | 8☐N/A | 9☐D/K | 99☐N/R |
| d. | Brother | 0☐No | 1☐Yes | 8☐N/A | 9☐D/K | 99☐N/R |
| e. | Grandparent | 0☐No | 1☐Yes | 8☐N/A | 9☐D/K | 99☐N/R |
| f. | Other | 0☐No | 1☐Yes | 8☐N/A | 9☐D/K | 99☐N/R |

If other, specify ________________________________________________

**2.4 Stroke**

| | | | | | | |
|---|---|---|---|---|---|---|
| a. | Mother | 0☐No | 1☐Yes | 8☐N/A | 9☐D/K | 99☐N/R |
| b. | Father | 0☐No | 1☐Yes | 8☐N/A | 9☐D/K | 99☐N/R |
| c. | Sister | 0☐No | 1☐Yes | 8☐N/A | 9☐D/K | 99☐N/R |
| d. | Brother | 0☐No | 1☐Yes | 8☐N/A | 9☐D/K | 99☐N/R |
| e. | Grandparent | 0☐No | 1☐Yes | 8☐N/A | 9☐D/K | 99☐N/R |
| f. | Other | 0☐No | 1☐Yes | 8☐N/A | 9☐D/K | 99☐N/R |

If other, specify ________________________________________________

2.5 **Obesity (very fat)**

| | | | | | | |
|---|---|---|---|---|---|---|
| a. | Mother | 0☐No | 1☐Yes | 8☐N/A | 9☐D/K | 99☐N/R |
| b. | Father | 0☐No | 1☐Yes | 8☐N/A | 9☐D/K | 99☐N/R |
| c. | Sister | 0☐No | 1☐Yes | 8☐N/A | 9☐D/K | 99☐N/R |
| d. | Brother | 0☐No | 1☐Yes | 8☐N/A | 9☐D/K | 99☐N/R |
| e. | Grandparent | 0☐No | 1☐Yes | 8☐N/A | 9☐D/K | 99☐N/R |
| f. | Other | 0☐No | 1☐Yes | 8☐N/A | 9☐D/K | 99☐N/R |

If other, specify ___________________________________________

**2.6 High cholesterol**

| | | | | | | |
|---|---|---|---|---|---|---|
| a. | Mother | 0☐No | 1☐Yes | 8☐N/A | 9☐D/K | 99☐N/R |
| b. | Father | 0☐No | 1☐Yes | 8☐N/A | 9☐D/K | 99☐N/R |
| c. | Sister | 0☐No | 1☐Yes | 8☐N/A | 9☐D/K | 99☐N/R |
| d. | Brother | 0☐No | 1☐Yes | 8☐N/A | 9☐D/K | 99☐N/R |
| e. | Grandparent | 0☐No | 1☐Yes | 8☐N/A | 9☐D/K | 99☐N/R |
| f. | Other | 0☐No | 1☐Yes | 8☐N/A | 9☐D/K | 99☐N/R |

If other, specify ___________________________________________

2.7 **Heart failure**

| | | | | | | |
|---|---|---|---|---|---|---|
| a. | Mother | 0☐No | 1☐Yes | 8☐N/A | 9☐D/K | 99☐N/R |
| b. | Father | 0☐No | 1☐Yes | 8☐N/A | 9☐D/K | 99☐N/R |
| c. | Sister | 0☐No | 1☐Yes | 8☐N/A | 9☐D/K | 99☐N/R |
| d. | Brother | 0☐No | 1☐Yes | 8☐N/A | 9☐D/K | 99☐N/R |
| e. | Grandparent | 0☐No | 1☐Yes | 8☐N/A | 9☐D/K | 99☐N/R |
| f. | Other | 0☐No | 1☐Yes | 8☐N/A | 9☐D/K | 99☐N/R |

If other, specify ___________________________________________

**2.8 Kidney failure**

| | | | | | | |
|---|---|---|---|---|---|---|
| a. | Mother | 0☐No | 1☐Yes | 8☐N/A | 9☐D/K | 99☐N/R |
| b. | Father | 0☐No | 1☐Yes | 8☐N/A | 9☐D/K | 99☐N/R |
| c. | Sister | 0☐No | 1☐Yes | 8☐N/A | 9☐D/K | 99☐N/R |
| d. | Brother | 0☐No | 1☐Yes | 8☐N/A | 9☐D/K | 99☐N/R |
| e. | Grandparent | 0☐No | 1☐Yes | 8☐N/A | 9☐D/K | 99☐N/R |
| f. | Other | 0☐No | 1☐Yes | 8☐N/A | 9☐D/K | 99☐N/R |

If other, specify ___________________________________________

2.9 **Asthma**

| | | | | | | |
|---|---|---|---|---|---|---|
| a. | Mother | 0□No | 1□Yes | 8□N/A | 9□D/K | 99□N/R |
| b. | Father | 0□No | 1□Yes | 8□N/A | 9□D/K | 99□N/R |
| c. | Sister | 0□No | 1□Yes | 8□N/A | 9□D/K | 99□N/R |
| d. | Brother | 0□No | 1□Yes | 8□N/A | 9□D/K | 99□N/R |
| e. | Grandparent | 0□No | 1□Yes | 8□N/A | 9□D/K | 99□N/R |
| f. | Other | 0□No | 1□Yes | 8□N/A | 9□D/K | 99□N/R |

If other, specify ____________________________________________

2.10 **Chronic Obstructive Airways Disease (COPD - bronchitis/emphysema)**

| | | | | | | |
|---|---|---|---|---|---|---|
| a. | Mother | 0□No | 1□Yes | 8□N/A | 9□D/K | 99□N/R |
| b. | Father | 0□No | 1□Yes | 8□N/A | 9□D/K | 99□N/R |
| c. | Sister | 0□No | 1□Yes | 8□N/A | 9□D/K | 99□N/R |
| d. | Brother | 0□No | 1□Yes | 8□N/A | 9□D/K | 99□N/R |
| e. | Grandparent | 0□No | 1□Yes | 8□N/A | 9□D/K | 99□N/R |
| f. | Other | 0□No | 1□Yes | 8□N/A | 9□D/K | 99□N/R |

If other, specify ____________________________________________

2.11 **Cancer**

| | | | | | | |
|---|---|---|---|---|---|---|
| a. | Mother | 0□No | 1□Yes | 8□N/A | 9□D/K | 99□N/R |
| b. | Father | 0□No | 1□Yes | 8□N/A | 9□D/K | 99□N/R |
| c. | Sister | 0□No | 1□Yes | 8□N/A | 9□D/K | 99□N/R |
| d. | Brother | 0□No | 1□Yes | 8□N/A | 9□D/K | 99□N/R |
| e. | Grandparent | 0□No | 1□Yes | 8□N/A | 9□D/K | 99□N/R |
| f. | Other | 0□No | 1□Yes | 8□N/A | 9□D/K | 99□N/R |

If other, specify ____________________________________________

If yes to any category above, state type of cancer and relative affected: ___________________________

______________________________________________________________________________

2.12 **Fractures (broken bones)**

| | | | | | | |
|---|---|---|---|---|---|---|
| a. | Mother | 0□No | 1□Yes | 8□N/A | 9□D/K | 99□N/R |
| b. | Father | 0□No | 1□Yes | 8□N/A | 9□D/K | 99□N/R |
| c. | Sister | 0□No | 1□Yes | 8□N/A | 9□D/K | 99□N/R |
| d. | Brother | 0□No | 1□Yes | 8□N/A | 9□D/K | 99□N/R |
| e. | Grandparent | 0□No | 1□Yes | 8□N/A | 9□D/K | 99□N/R |
| f. | Other | 0□No | 1□Yes | 8□N/A | 9□D/K | 99□N/R |

If other, specify ____________________________________________

2.13 **Mental Health Disorders** (e.g. anxiety, depression, schizophrenia)

| | | | | | | |
|---|---|---|---|---|---|---|
| a. | Mother | 0□No | 1□Yes | 8□N/A | 9□D/K | 99□N/R |
| b. | Father | 0□No | 1□Yes | 8□N/A | 9□D/K | 99□N/R |
| c. | Sister | 0□No | 1□Yes | 8□N/A | 9□D/K | 99□N/R |
| d. | Brother | 0□No | 1□Yes | 8□N/A | 9□D/K | 99□N/R |
| e. | Grandparent | 0□No | 1□Yes | 8□N/A | 9□D/K | 99□N/R |
| f. | Other | 0□No | 1□Yes | 8□N/A | 9□D/K | 99□N/R |

If other, specify ______________________________________________

2.14 Has anyone in your family died suddenly ***(unexpected death except motor vehicle accident or other accidental or violent death)***

0□No 1□Yes 9□D/K 99□N/R

**If yes,** give relationship, age, and cause of death if known? ***If NO go to section 3***

a. **Father** 0□No 1□Yes 9□D/K 99□N/R

Cause of death (if known) ______________________________ Age □□

ICD Cod:□□□.□

b. **Mother** 0□No 1□Yes 9□D/K 99□N/R

Cause of death (if known) ______________________________Age □□

ICD Code:□□□.□

c. **Brother/Sister** 0□No 1□Yes 9□D/K 99□N/R

Cause of death (if known) _______________________________Age □□

ICD Code:□□□.□

d. **Grandparent** 0□No 1□Yes 9□D/K 99□N/R

Cause of death (if known) _______________________________Age □□

ICD Code: □□□.□

e. **Other**, specify _________________________________________________

Cause of death (if known) _______________________________Age □□

## SECTION 3: MEDICAL HISTORY

*I am going to ask you some questions about your health and any sicknesses you might have had in the past.*

*Think carefully before you answer because it is important that we get very accurate information.*

**(DK= Don't Know. NR= No Response)**

3.1 Have you ever been diagnosed or treated for any of the following medical conditions?

| | | | | |
|---|---|---|---|---|
| a. Heart disease | 0□ NO | 1□ YES | 9□ D/K | 99□N/R |
| b. Rheumatic fever | 0□ NO | 1□ YES | 9□ D/K | 99□N/R |
| c. Diabetes mellitus (sugar) | 0□ NO | 1□ YES | 9□ D/K | 99□N/R |
| d. Sickle cell disease | 0□ NO | 1□ YES | 9□ D/K | 99□N/R |
| e. Sickle cell trait | 0□ NO | 1□ YES | 9□ D/K | 99□N/R |
| f. Stroke | 0□ NO | 1□ YES | 9□ D/K | 99□N/R |
| g. High blood pressure | 0□ NO | 1□ YES | 9□ D/K | 99□N/R |
| h. High cholesterol | 0□ NO | 1□ YES | 9□ D/K | 99□N/R |
| i. Obesity / Overweight | 0□ NO | 1□ YES | 9□ D/K | 99□N/R |
| j. Asthma / Wheezing | 0□ NO | 1□ YES | 9□ D/K | 99□N/R |
| k. Bronchitis | 0□ NO | 1□ YES | 9□ D/K | 99□N/R |
| l. Kidney disease | 0□ NO | 1□ YES | 9□ D/K | 99□N/R |
| m. Sexually trans. disease | 0□ NO | 1□ YES | 9□ D/K | 99□N/R |
| n. Pneumonia / Bronchitis | 0□ NO | 1□ YES | 9□ D/K | 99□N/R |
| o. Cerebral palsy | 0□ NO | 1□ YES | 9□ D/K | 99□N/R |
| p. Eczema | 0□ NO | 1□ YES | 9□ D/K | 99□N/R |
| q. Muscle dystrophy | 0□ NO | 1□ YES | 9□ D/K | 99□N/R |
| r. Paralysis /Weak legs | 0□ NO | 1□ YES | 9□ D/K | 99□N/R |
| s. Abnormalities of the spine (e.g. spina bifida, scoliosis) | 0□ NO | 1□ YES | 9□ D/K | 99□N/R |
| t. Arthritis | 0□ NO | 1□ YES | 9□ D/K | 99□N/R |
| u. Thyroid problems | 0□ NO | 1□ YES | 9□ D/K | 99□N/R |
| v. Epilepsy/seizures | 0□ NO | 1□ YES | 9□ D/K | 99□N/R |
| w. Broken bones/fracture | 0□ NO | 1□ YES | 9□ D/K | 99□N/R |

If yes, specify bone and where treated: ____________________________________________

3.2a Are you taking any medication / medicines / tablets on a regular basis?

0□NO 1□YES ***(If no, go to question 3.3)***

3.2b. If yes please give names and the condition for which the medication is being taken:

______________________________

______________________________

______________________________

3.3a Have you been prescribed any other medication that you should take regularly, but are not taking? 0□ NO 1□ YES ***(If no, go to question 3.4)***

3.3b. If yes, please give names of medications and the condition for which the medication was prescribed: ______________________________

______________________________

______________________________

3.4a. Do you currently take multivitamin supplements? 0□ NO 1□ YES 9□D/K 99□N/R ***(If no, go to question 3.4d)***

3.4b. How many tablets do you take per week? □□Tablets

3.4c. For how long have you been taking these tablets? □□ Months

3.4d Do you currently take Iron (tablets / liquid)? 0□No 1□yes 9□D/K 99□N/R

3.5 Do you take herbal supplements?

0□No 1□yes 9□D/K 99□N/R

If yes, specify ______________

3.6 Have you had or been treated for any infections or inflammatory condition (e.g. flu-like illness pneumonia, arthritis etc) in the last six months?

0□ NO 1□ YES 9□ D/K 99□N/R ***(If no, go to question 3.7)***

3.6b. If yes, give details: Name of condition______________________

Approximate date __________ Treatment received ______________

Approximate date of recovery __________

3.7 Have you ever had any surgery / operations done at any time in the past?

0□ NO 1□ YES 9□D/K 99□ N/R **(If no, go to question 3.8)**

a. If yes, specify the type of operation and when it was done. ____________________________

_______________________________________________________________

3.8. Approximately how often do you usually see a doctor?

0□ < once per year 1□ 1-2 times/year 2□ 3-4 times/year

4□ >four times/year 9□ D/K 99□ N/R

3.9. Where do you usually go to see the doctor? (select only one response)

1□ hospital casualty / emergency room 2□ hospital clinic 3□ health centre

4□ private doctor – general practitioner 5□ private doctor – specialist

6□ other, specify ___________________

3.10a Were you interviewed in the last follow up? 0□No 1□Yes 9□D/K 99□N/R

3.10b If yes, have you been admitted in hospital (stayed overnight or longer), since the last time you were interviewed or in the last 4 years?

0□NO 1□ YES 9□ D/K 99□ N/R **(If no, go to question 3.11)**

3.10c If no, have you been admitted in hospital (stayed overnight or longer), in the last 4 years?

0□NO 1□ YES 9□ D/K 99□ N/R **(If no, go to question 3.11)**

3.10d If yes to 3.10b or c, list all the admissions and the reason you were admitted in the box below.

**(If more than 5 hospitalizations fill out a separate form)**

| Admission # | Age at admission | No. of nights | Reason for admission | Hospital name |
|---|---|---|---|---|
| 1 | | | | |
| 2 | | | | |
| 3 | | | | |
| 4 | | | | |
| 5 | | | | |

3.10e If there are more than 5 admissions give approximate total □□

3.11 ***(For females only)***

a. When was your last menstrual period?

1□ <1 Month ago 2□ 1-2 Month ago 3□>2Month ago 9□ D/K 99□ N/R

b. Have you ever been pregnant? 0□NO 1□YES 9□ D/K 99□ N/R

***(If no, go to section3.12)***

c. How many times have you been pregnant? □□

d. Have you had any miscarriages? 0□NO 1□YES 9□ D/K 99□ N/R

If yes how many? ________________________

e. How many children do you have? □□ 9□ D/K 99□ N/R

f. How old are the children? _________________________

g. Did you breastfeed the child/children? 0□NO 1□YES 9□ D/K 99□ N/R

h. Did you take calcium supplements during the pregnancy?

0□NO 1□YES 9□ D/K 99□ N/R

**(Males and females)**

3.12 When was the last time you were seen by a dentist?

0□ NEVER 1□ <1 Year ago 2□ 1-2Years ago 3□ > 2Years ago

9□ D/K 99□ N/R

## SECTION4: PHYSICAL ACTIVITY, SOCIAL HABITS, AND EXPOSURE TO INJURY/VIOLENCE

***For questions 4.1-4.5, give your answer using the stated options.***

***ACTIVITY***

4.1. How do you rate your level of physical activity?

1□ very active 2□ moderately active

3□ occasionally active 4□ inactive 9□I don't know

4.2. Over **the last seven days**, how many days did you play an active sport?

(e.g. football, cricket, netball, swimming)

0□ Never 1□ 1-2 times 2□ 3-4 times 3□ 5-6 times 4□ everyday 9□ D/K

99□N/R

4.3. On average, how many hours do you spend playing a sport each week?

1□ None 2□ less than 1hr 3□ 1-2hrs 4□ 3-4hrs 5□ 5-10hrs 6□ 11-20hrs

7□ >20hrs

4.4. Over **the last seven days**, on how many days did you do other exercise (e.g. brisk walking, jogging, lifting weights, dance classes, or workout at a gym)?

1□ Never 2□ 1-2 times 3□ 3-4 times 4□ 5-6 times 5□ everyday

9 □DK 99□NR

4.5. On average, how many hours do you spend each week doing the exercise stated in 4.4?

1□ None 2□ less than 1hr 3□ 1-2hrs 4□ 3-4hrs 5□ 5-10hrs 6□ 11-20hrs

7□ >20hrs 9 □DK 99□NR

4.6 At what grade did you stop having physical education (PE) / games classes at school? □□

4.6a How old were you when you stopped doing physical Exercise at school?

□□ Yrs 9□□DK 99□□NR

4.6b Did you represent your school in any sport?

0□NO 1□YES 9□DK 99□NR

4.6c. Do you watch television? 0□NO 1□YES **(If no, skip to question 4.7)**

4.6d. How many hours of television do you watch per day during the week (Monday-Friday) □□

4.6e. How may hours of television do you watch in total on Saturday & Sunday (weekends) □□

***Tobacco, alcohol and other drugs***

4.7. Have you ever smoked a cigarette or other form of tobacco (cigar or pipe)?

0☐ NO 1☐ YES 9☐D/K 99☐N/R

**(If you have never smoked tobacco go to question 4.8)**

4.7a. How old were you when you smoked a cigarette or cigar for the first time?

☐☐yrs 9☐D/K 99☐N/R

4.7b. Do you currently smoke? 0 ☐NO 1 ☐YES

4.7c. In the past month, on how many days did you smoke cigarettes?

☐☐days 9☐D/K 99☐N/R

4.7d.On average, how many cigarettes do you smoke (or used to smoke) per week? ☐☐☐cigarettes

8☐N/A, specify ____________________________________ 9☐D/K 99☐N/R

4.7e. Have you smoked more than 100 cigarettes in your lifetime?

0☐NO 1☐YES 9☐D/K 99☐N/R

4.8. Have you ever smoked a ganja / marijuana / weed?

0☐NO 1☐YES 9☐D/K 99☐N/R **(If you have never smoked ganja go to question 4.9)**

4.8a How old were you when you smoked ganja for the first time? ☐☐yrs 9☐D/K 99☐N/R

4.8b.In the past month, on how many days did you smoke ganja?

0☐ None 1☐ Less than once/week 3☐ 1-2days /week 4☐ 3-4days/week

5☐ 5 or more days/week 9☐DK 99☐NR

4.8c.On average, how many spliffs do you smoke (or used to smoke) per week?

☐☐spliffs 9☐D/K 99☐N/R

4.9. Do you use ganja in any form apart from smoking? **(If no, go to question 4.10)**
0☐ NO 1☐ YES 9☐ DK 99☐ NR

4.9a.What are the other forms? ***PROMPT*** **MULITPLE RESPONSES ALLOWED**

1☐ Tea 2☐ Used in cooking 3☐Other, specify________________ 9☐D/K 99☐N/R

4.10. Have you ever had alcohol to drink? 1☐ YES 0☐ NO 9☐D/K 99☐N/R

(Includes beer, wine, rum, brandy etc, but no alcohol in cakes or drinks such as sorrel)

**(If you have never had alcohol, go to question 4.11)**

4.10a. How old were you when you drank alcohol for the first time? ☐☐yrs 9☐D/K 99☐N/R

4.10b. In the past month, on how many days did you drink alcohol?

0☐ None 1☐ < once/wk 2☐ 1-2days /wk 3☐ 3-4days/wk

4☐ ≥5days/wk 9☐D/K 99☐N/R

4.10c. On average how many drinks do you have per week? ☐☐☐

8☐N/A, specify ________________________________ 9☐D/K 99☐N/R

(**One drink of alcohol is any of: 1 bottle (12 oz) beer/stout; 1 glass (4 oz) wine; 1 drink (1 oz) spirit)**

4.10d. ***(Males only)*** Have you ever had more than 5 drinks at one session?

0☐NO 1☐YES 9☐D/K 99☐N/R

4.10e. ***(Females)*** Have you ever had more than 4 drinks at one session?

0☐NO 1☐YES 9☐D/K 99☐N/R

4.10f. How often do you have a drinking binge?

**(i.e. more than 5 drinks/session for males or 4 drinks/session for females)**

1☐<once/mth 2☐1-2 times/mth 3☐3-4 times/mth 4☐≥5 times/mth 9☐D/K 99☐N/R

4.10g. What is the maximum number of drinks you have had in one day? ☐☐ 9☐D/K 99☐N/R

4.10f. What type of alcoholic drink do you have most of the time? (select only one)

1☐beer 2☐stout 3☐wine 4☐rum 5☐brandy

6☐vodka 7☐other, specify _________ 9☐DK 99☐NR

4.11 Have you ever used any of the following drugs?

a. Cocaine 0☐NO 1☐ YES 9☐DK 99☐NR

b. Crack ☐NO 1☐ YES 9☐DK 99☐NR

c. heroin, morphine ☐NO 1☐ YES 9☐DK 99☐NR

4.11a. Have you used any of the following drugs in the past month?

a. Cocaine 0☐NO 1☐ YES 9☐DK 99☐NR

b. Crack 0☐NO 1☐ YES 9☐DK 99☐NR

c. heroin, morphine 0☐NO 1☐ YES 9☐DK 99☐NR

4.11b Have you ever injected drugs* into any part of your body with a needle? (* other than drugs prescribed by a doctor to treat a medical condition)

0☐NO 1☐ YES 9☐DK 99☐NR **( If no, go to question 4.12)**

If yes, give the name of the drug(s): ________________________________

4.11c If yes to 4.11b, have you ever shared needles used to inject drugs with another person?

0☐NO 1☐ YES 9☐DK 99☐NR

## ***INJURY & VIOLENCE***

4.12. When traveling in a motor vehicle, do you use the seat belt?

0□never 1□infrequently(rarely) 2□frequently(most times) 3□always 99□NR

4.12a. When riding a motorcycle or pedal cycle, do you use a protective helmet?

0□never 1□infrequently(rarely) 2□frequently(most times) 3□always 99□NR

4.12b. In the past three years, have you been involved in any motor vehicle accident?

0□NO 1□ YES 9□DK 99□NR

4.12c. In the past three years, have you been injured in a motor vehicle accident?

0□NO 1□ YES 9□DK 99□NR

4.12d. In the past three years, have you been involved in any fights or been attacked by anyone?

0□NO 1□ YES 9□DK 99□NR

4.12e. In the past three years, have you been injured in a fight or in an attack by anyone?

0□NO 1□ YES 9□DK 99□NR

4.12f Do you carry any object or weapon to protect yourself from being attacked by other persons?

0□NO 1□ YES 9□DK 99□NR

## Section 5: Socioeconomic Status

5.1. Who do you live with? (Choose only one) 1☐MOTHER 2☐FATHER
3☐BOTH PARENTS 4☐SELF 5☐SPOUSE
7☐OTHER, specify ______________________ 9☐DK 99☐NR

5.2. Who is mainly responsible to provide for you financially?
1☐ father 2☐ mother 3☐both father & mother 4☐ guardian 5☐ self
6☐ spouse 7☐ other, specify __________ 9☐DK 99☐NR

5.3. What is the occupation of your father? ______________________ 9☐DK 99☐NR

5.4. What is the occupation of your mother? ______________________ 9☐DK 99☐NR

5.5. If your primary financial support is not your mother or father, what is the occupation of this person?
______________________________________ 9☐DK 99☐NR

5.6. What is the highest education level of your mother, father or guardian/financial provider?

**a. Mother**: 0☐ never attended school 1☐basic school only 2☐ primary/all-age
3☐ secondary/high/technical 4☐ Tertiary- College/University 9☐ DK 99☐ NR

**b. Father**: 0☐ never attended school 1☐basic school only 2☐ primary/all-age
3☐ secondary/high/technical 4☐ Tertiary- College/University 9☐ DK 99☐ NR

**c. guardian**: 0☐ never attended school 1☐basic school only 2☐ primary/all-age
3☐ secondary/high/technical 4☐ Tertiary- College/University 8☐N/A 9☐ DK 99☐ NR

5.7. Who is considered the head of your household (major wage earner)?
1☐ father 2☐ mother 3☐ guardian 4 ☐ self 5☐ spouse
7☐ other, specify __________ 9☐DK 99☐NR

5.8. What is the occupation of your household head (major wage earner)?
______________________ 9☐Don't Know

5.9. Are you currently working for pay? 0☐NO 1☐YES 9☐DK 99☐NR
If yes, what type of work do you do? ______________________________

5.10. Have you worked for pay in the past? 0☐NO 1☐YES 9☐DK 99☐NR
If yes, what type of work did you do? ______________________________

5.11. Are you now attending school, college or university?

0□NO 1□YES 9□DK 99□NR

If yes,

a. State institution ______________________________

b. Who is paying for your studies? 1□ father 2□ mother 3□ both mother & father
4□ guardian 5□ other, specify ____________

5.12. Do you have additional sources of income?

0□NO 1□YES 9□DK 99□NR

a. If yes, state source ______________________________

5.13. What is the average combined monthly income of your household (mother, father, guardian, self, etc)?

1 □ Less than J$ 4,000
2 □ J$ 4,001 – 10,000
3 □ J$ 10,001 – 20,000
4 □ J$ 20,001 – 40,000
5 □ J$ 40,001 – 80,000
6 □ >J$ 80,000
9□ DK
99 □ NR/Refused

5.14. What is your current monthly income?

1 □ Less than J$ 4,000
2 □ J$ 4,001 – 10,000
3 □ J$ 10,001 – 20,000
4 □ J$ 20,001 – 40,000
5 □ J$ 40,001 – 80,000
6 □ >J$ 80,000
7 □ not working
9 □ DK
99□ NR/Refused

5.15. What type of house do you live in?

1□ concrete 2□ board/wood 3□ other, specify ______________________

9□DK 99□NR

5.16. How many bedrooms (used for sleeping) are in your house? □□ 9□DK 99□NR

5.17. How many other rooms (living room or other habitable room) are in your house?

□□ 9□DK 99□NR

5.18. Is the house that you live in rented or owned by the family

1□owned by parent(s)/guardian 2□rented 3□family home (owned by other family)

4□other, specify____________________________________ 9□DK 99□NR

5.19. How many adults (≥18yrs) including yourself, live in the same house with you? □□□

5.20. How many children (under 18 yrs old) live in the same house with you? ________________

5.21. Which of the following do you have working in your home?

| | | | | |
|---|---|---|---|---|
| 1. Television set | 0□NO | 1□YES | 9□D/K | 99□N/R |
| 2. Cable/Satellite connection | 0□NO | 1□YES | 9□D/K | 99□N/R |
| 3. Gas / electric stove | 0□NO | 1□YES | 9□D/K | 99□N/R |
| 4. Refrigerator | 0□NO | 1□YES | 9□D/K | 99□N/R |
| 5. Freezer | 0□NO | 1□YES | 9□D/K | 99□N/R |
| 6. Living room set | 0□NO | 1□YES | 9□D/K | 99□N/R |
| 7. Stereo equipment | 0□NO | 1□YES | 9□D/K | 99□N/R |
| 8. Washing machine | 0□NO | 1□YES | 9□D/K | 99□N/R |
| 9. Cars or other vehicles | 0□NO | 1□YES | 9□D/K | 99□N/R |
| 10. Telephone | 0□NO | 1□YES | 9□D/K | 99□N/R |
| 11. VCR (video player) | 0□NO | 1□YES | 9□D/K | 99□N/R |
| 12. DVD/CD | 0□NO | 1□YES | 9□D/K | 99□N/R |
| 13. Computer | 0□NO | 1□YES | 9□D/K | 99□N/R |
| 14. Internet connection | 0□NO | 1□YES | 9□D/K | 99□N/R |
| 15. Radio/cassette player | 0□NO | 1□YES | 9□D/K | 99□N/R |
| 16. Microwave oven | 0□NO | 1□YES | 9□D/K | 99□N/R |
| 17. Air-conditioning | 0□NO | 1□YES | 9□D/K | 99□N/R |

5.22. What type of toilet facilities do you use at home?

1 □ none
2 □ hole in the ground
3 □ pit latrine, shared
4 □ pit latrine, unshared
5 □ water closet (flush type), shared
6 □ water closet (flush type), unshared
7 □ other, specify _____________
9 □ DK
99 □ NR

5.22. What is your source of water?

1 □ stream/river
2 □ catchment (drum etc.)
3 □ standpipe
4 □ piped, in yard
5 □ piped, in house
6 □ other, specify ___________________
9 □ DK
99 □ NR

5.23. What is the usual source of light in your home?

1☐electricity 2☐kerosene lamp 3☐candle 4☐other, specify ____________

9 ☐ DK 99 ☐ NR

5.24. What type of cooking facilities do you have at home?

1☐wood 2☐coal stove 3☐kerosene stove 4☐gas stove / electric stove

5☐other, specify ________________ 9☐DK 99☐NR

5.25. How many **miles walking distance** are the following from your house?

| | | | | | | |
|---|---|---|---|---|---|---|
| a. Public Transportation | 1☐<1 | 2☐1-2 | 3☐3-4 | 4☐5-10 | 5☐>10 | 9☐Not Known |
| b. Bank | 1☐<1 | 2☐1-2 | 3☐3-4 | 4☐5-10 | 5☐>10 | 9☐Not Known |
| c. Market/Supermarket | 1☐<1 | 2☐1-2 | 3☐3-4 | 4☐5-10 | 5☐>10 | 9☐Not Known |
| d. Primary school | 1☐<1 | 2☐1-2 | 3☐3-4 | 4☐5-10 | 5☐>10 | 9☐Not Known |
| e. Post Office | 1☐<1 | 2☐1-2 | 3☐3-4 | 4☐5-10 | 5☐>10 | 9☐Not Known |
| f. Health Centre/Doctor | 1☐<1 | 2☐1-2 | 3☐3-4 | 4☐5-10 | 5☐>10 | 9☐Not Known |
| g. Police Station | 1☐<1 | 2☐1-2 | 3☐3-4 | 4☐5-10 | 5☐>10 | 9☐Not Known |

## Attitudes towards Fatness

*The table below contains statements about fatness. Kindly state if you agree or disagree with each of the statement below.*

| | **Strongly disagree 1** | **Disagree 2** | **Agree 3** | **Strongly agree 4** |
|---|---|---|---|---|
| Men love fat women. | | | | |
| Most fat individuals have little ambition | | | | |
| I would prefer a normal weight person rather than a fat person to be my friend. | | | | |
| Slim people are healthier than fat persons | | | | |
| Fat people are lazy. | | | | |
| Fat individuals exercise less than normal weight persons. | | | | |
| Slim people are more popular than fat people. | | | | |
| Fatness in older persons is more acceptable than in younger persons. | | | | |
| Most fat adolescents play fewer active games than normal weight adolescents. | | | | |
| Fat persons are greedy. | | | | |
| Fat persons eat a lot of fatty foods. | | | | |
| Less emphasis should be placed on fatness as a health problem. | | | | |

2. How satisfied are you with your weight?
   1. [ ] Satisfied
   2. [ ] Not satisfied

3. In relation to your height, do you think you are ….
   1. [ ] underweight 2. [ ] right weight
   3. [ ] a little overweight 4. [ ] very overweight or obese.

4. At present, are you doing anything to change your weight?
   1. [ ] I am trying to gain weight
   2. [ ] I am trying to lose weight
   3. [ ] I am not doing anything about my weight.

# SECTION 6: DIETARY HABITS & BODY IMAGE/ PERCEPTION

6.1 Are you on a special diet whether for religious or medical or other reasons?
0□NO 1□YES 9□DK 99□NR

6.1a If yes: **(multiple responses allowed)**

| | | | |
|---|---|---|---|
| Vegetarian | 0□NO | 1□YES | 99□NR |
| Weight loss | 0□NO | 1□YES | 99□NR |
| Weight gain | 0□NO | 1□YES | 99□NR |
| Diabetic | 0□NO | 1□YES | 99□NR |
| Low salt | 0□NO | 1□YES | 99□NR |
| Low fat | 0□NO | 1□YES | 99□NR |
| Low cholesterol | 0□NO | 1□YES | 99□NR |
| Other | 0□NO | 1□YES | 99□NR |

6.1b. Which is the most important source of information on nutrition to you? **(Select one only)**

| | |
|---|---|
| Radio | 1□ |
| T.V. | 2□ |
| Brochures/Pamphlets | 3□ |
| Doctor/Nurse | 4□ |
| Dietitian/Nutritionist | 5□ |
| Health related organization | 6□ |
| Friend/relative | 7□ |
| Don't Know | 9□ |
| No Response | 99□ |

6.2 How many times per week do you eat at a fast food outlet?
0□Never 1□1-2 times/week 2□3-4 times/week 3□5-6 times/week 4□everyday
9□DK 99□NR

*I am now going to show you some drawings of different body types.*

***In Women***

6.3a. Which images do you think correspond to a normal body size and shape? □ - □
6.3b Which body image do you think people find most attractive? □
6.3c.Which body image do you think is most healthy? □
6.3d Which of these images do you think corresponds most to your current body size and shape? □
6.3e Which of these images is closest to the size and shape you would most like to be? □

***In Men***

6.3f Which of these images do you think correspond to a normal body size and shape? □ - □
6.3g.Which body image do you think people find most attractive? □
6.3h.Which body image do you think is most healthy? □
6.3i Which of these images do you think corresponds most to your current body size and shape? □
6.3j Which of these images is closest to the size and shape you would most like to be? □

**Duration of Interviews** □**hrs** □□**min**

# SECTION 7: BODY MEASUREMENTS

*NAME OF PARTICIPANT*: ______________________________________

Now I am going to measure your height, weight, and waist and hip measurements. I will explain each one as we do it.

| | |
|---|---|
| 6.1 WEIGHT<br><br>RECORD SCALE IDENTIFICATION NUMBER | \|____\|____\|____\| . \|____\| Kg<br><br>\|____\|____\| |
| 6.2 HEIGHT | \|____\|____\|____\| . \|____\| cm |
| 6.3 WAIST CIRCUMFERENCE<br>**(Mid-point between lowest rib and iliac crest)**<br><br>Over what clothing was the measurement taken? | 1. \|____\|____\|____\| . \|____\| cm<br>2. \|____\|____\|____\| . \|____\| cm<br>3. \|____\|____\|____\| . \|____\| cm<br><br>0 [ ] No clothing: skin<br>1 [ ] Shirt or dress<br>2 [ ] Trousers only<br>3 [ ] Shirt & trousers |
| 6.4 BUTTOCKS (HIP) CIRCUMFERENCE<br><br>**(At the level of greater trochanter)**<br><br>Over what clothing was the measurement taken? | 1. \|____\|____\|____\| . \|____\| cm<br>2. \|____\|____\|____\| . \|____\| cm<br>3. \|____\|____\|____\| . \|____\| cm<br><br>0 [ ] No clothing: skin<br>1 [ ] Shirt or dress<br>2 [ ] Trousers only<br>3 [ ] Shirt & trousers |
| Thickness of upper body covering: | 0 [ ] None 1 [ ] Thin<br>2 [ ] Thick |
| Thickness of lower body covering: | 0 [ ] None 1 [ ] Thin<br>2 [ ] Thick |

## 6.5 Skin-fold thickness

| | | | | | |
|---|---|---|---|---|---|
| a. | Triceps | □□□mm | b. | Biceps | □□□mm |
| c. | Subscapular | □□□mm | d. | suprailiac | □□□mm |

## SECTION 8: FASTING GLUCOSE TEST

*NAME OF PARTICIPANT*: ________________________________________

1. What time and date did you last eat? TIME: |____|____| : |____|____| 0 [ ] AM
1 [ ] PM
DATE: _____/_____/_____
DY MO YR

2. What time and date did you last have something other than water to drink? TIME: |____|____| : |____|____| 0 [ ] AM
1 [ ] PM
DATE: _____/_____/_____
DY / MO / YR

3. What time and date did you last smoke? TIME: |____|____| : |____|____| 0 [ ] AM
1 [ ] PM
DATE: _____/_____/_____
DY MO YR

Date the blood was drawn: DATE: _____/_____/_____
DY MO YR

Time of fasting sample: |____|____| : |____|____| 0 [ ] AM
1 [ ] PM

Case ID# ☐☐☐☐☐☐☐☐☐ Interviewer's ID # ☐☐☐☐



## SECTION 9: PULSE AND BLOOD PRESSURE MEASUREMENT

*NAME OF PARTICIPANT:* ___________________________________________

*Now I will explain the procedure for measuring your pulse and blood pressure. It is important that you remain relaxed and seated for the measurement which will take about 15 minutes. Please do not cross your feet or legs during the measurements. I will wrap the blood pressure cuff around your arm, take your pulse and then inflate the cuff. You will feel a sensation of pressure on your arm when the cuff is inflated. I will be inflating the cuff a maximum of 5 times. While I am measuring your blood pressure, it is best if we do not talk. If you have any questions, I will be happy to answer them for you before or after the measurement is taken. I will tell you the results of the measurements afterward.*

**Record Baumanometer Identification #** ☐☐☐☐

| | |
|---|---|
| 1. Have you had any food, alcohol, coffee or cigarettes within the last 30 minutes? | Food: 0 [ ] N 1 [ ] Y<br>Alcohol: 0 [ ] N 1 [ ] Y<br>Coffee: 0 [ ] N 1 [ ] Y<br>Cigarettes: 0 [ ] N 1 [ ] Y |
| 2. Arm circumference:<br>*(Mid-point between inferior border of the olecranon & the lateral projection of acromion)* | \|____\|____\| . \|____\| |
| 3. Cuff size selected: | 0 [ ] Small adult 9 (17 – 25 cm)<br>1 [ ] Adult (25 – 35 cm)<br>2 [ ] Large (31 – 40 cm)<br>3 [ ] Thigh (38 – 50 cm) |
| 4. Arm selected:<br>(Preferably right arm) | 0 [ ] Right<br>1 [ ] Left ________________________<br>Reason |
| 5. Pulse rate for 30 seconds: | \|____\|____\| |
| 6. Pulse regular? | 1 [ ] Yes<br>1 [ ] No |
| 7. Pulse Obliteration Pressure (POP): | \|____\|____\|____\| |
| 8. Maximum inflation level: POP + 30 mmHg= | \|____\|____\|____\| |
| 9. First blood pressure measurement:<br>0 [ ] BP refused – Reason : _________________<br>1 [ ] BP not done – Reason: ________________ | \|____\|____\|____\| / \|____\|____\|____\|<br>SBP DBP |
| 10. Pulse rate for 30 seconds: | \|____\|____\|____\| |
| 11. Second blood pressure measurement: | \|____\|____\|____\| / \|____\|____\|____\|<br>SBP DBP |
| 12. Pulse rate for 30 seconds: | \|____\|____\|____\| |
| 13. Third blood pressure measurement: | \|____\|____\|____\| / \|____\|____\|____\|<br>SBP DBP |

# SECTION 10: URINE COLLECTION DATA

*NAME OF PARTICIPANT*: ______________________________________

| | | |
|---|---|---|
| 9.1 | Time of bladder first emptying / starting time of timed collection | □□: □□ AM |
| 9.2 | Time of second micturition / end of timed collection | □□: □□ AM/PM (circle the correct option) |
| 9.3 | Time of final collection if different from 2nd | □□: □□ AM/PM |
| 9.4 | Volume of urine passed | □□□□ml |
| 9.5 | Volume of urine collected for processing | □□□□ml |

# SECTION 11: LABORATORY RESULTS

*NAME OF PARTICIPANT*: ________________________________________

| | |
|---|---|
| 10.1. Fasting blood glucose | □□.□ mmol/L |
| 10.2. Glycosylated haemoglobin | □□.□ % |
| 10.3. Total cholesterol | □□.□ mmol/L |
| 10.4. LDL cholesterol | □□.□ mmol/L |
| 10.5. HDL cholesterol | □□.□ mmol/L |
| 10.6. Triglycerides | □□.□ mmol/L |
| 10.7. Fasting insulin | □□.□ |
| 10.8. Haemoglobin | □□.□ x $10^9$/L |
| 10.9. Packed cell volume | □□.□ % |
| 10.10. White cell count | □□.□ x $10^9$/L |
| 10.11. Platelet count | □□□.□ x $10^9$/L |
| 10.12. Sodium | □□.□ mmol/L |
| 10.13. Potassium | □□.□ mmol/L |
| 10.14. Chloride | □□.□ mmol/L |
| 10.15. Bicarbonate | □□.□ mmol/L |
| 10.16. Urea | □□.□ mmol/L |
| 10.17. Creatinine | □□.□ mmol/L |
| 10.18. high sensitivity C-reactive protein | □□.□ |

## SECTION 11Cont: LABORATORY RESULTS

*NAME OF PARTICIPANT*: ________________________________________

10.19. Serum Ferritin □□.□

10.20. Apolipoprotein B-100 □□.□

10.21 Interleukin 6

10.22 Soluble intercellular adhesion molecule (type 1) □□.□

10.22 Homocysteine □□.□

10.23 Microalbuminuria □□□

10.24 Urinary sodium □□□

## SECTION 12: BONE MINERAL DENSITY DATA

11.1. NAME: ______________________________________________

11.2 Sex: □Male □Female

**11.3. Bone Mineral Density result**

**(1) T-score**

**a. Right Heel** □□.□

**b. Left Heel** □□.□

**(2) Stiffness Index**

**a. Right Heel** _______________

**b. Left Heel** _______________
