# Supplementary material for: Factors associated with elevated blood pressure or hypertension in Afro-Caribbean youth: a cross-sectional study
Source: PeerJ. 2018 Feb 13;6:e4385. doi: 10.7717/peerj.4385 (PMC5815333; doi:10.7717/peerj.4385)
Supplement: Supplemental Information 4 [file peerj-06-4385-s004.pdf]

num

```
paricipant number
-------------------------------------------------------------------------------------------------

                  type:  numeric (float)

                 range:  [1,902]                      units:  1
         unique values:  898                      missing .:  0/898

                  mean:   452.213
              std. dev:   260.802

           percentiles:        10%       25%       50%       75%       90%
                                90       226     453.5       678       813

-------------------------------------------------------------------------------------------------
```

q112age

```
age at last birthday
-------------------------------------------------------------------------------------------------

                  type:  numeric (byte)

                 range:  [18,20]                      units:  1
         unique values:  3                        missing .:  0/898

            tabulation:  Freq.  Value
                           283  18
                           525  19
                            90  20

-------------------------------------------------------------------------------------------------
```

q71wgt

```
weight (kg)
----------------------------------------------------------------------------------------------------------
                  type:  numeric (float)

                 range:  [38.1,140]                   units:  .1
         unique values:  436                      missing .:  0/898

                  mean:   66.3664
              std. dev:   15.5442

           percentiles:        10%       25%       50%       75%       90%
                              49.3      55.7      64.4      73.4      84.7

----------------------------------------------------------------------------------------------------------
```

q72hgt

```
height (cm)
----------------------------------------------------------------------------------------------------------

                  type:  numeric (float)

                 range:  [141,208]                    units:  .1
         unique values:  325                      missing .:  0/898

                  mean:    169.61
              std. dev:   9.10536

           percentiles:        10%       25%       50%       75%       90%
                             157.8     162.5    169.45     176.4     181.7

----------------------------------------------------------------------------------------------------------
```

q1117cre

```
creatinine (mmol/l)
-----------------------------------------------------------------------------------------------

                  type:  numeric (float)

                 range:  [5.2,512]                    units:  .1
         unique values:  90                       missing .:  45/898

                  mean:   67.6558
              std. dev:   24.6935

           percentiles:        10%       25%       50%       75%       90%
                              42.1      53.9      66.9      82.4      92.1

-----------------------------------------------------------------------------------------------
```

waist_idf

```
central obesity (IDF cutpoints)
-----------------------------------------------------------------------------------------------

                  type:  numeric (float)
                 label:  true

                 range:  [0,1]                        units:  1
         unique values:  2                        missing .:  1/898

            tabulation:  Freq.   Numeric  Label
                           757         0  No
                           140         1  Yes
                             1         .

-----------------------------------------------------------------------------------------------
```

lowhdl

```
Low HDL
-----------------------------------------------------------------------------------------------

                  type:  numeric (float)
                 label:  true

                 range:  [0,1]                        units:  1
         unique values:  2                        missing .:  50/898

            tabulation:  Freq.   Numeric  Label
                           449         0  No
                           399         1  Yes
                            50         .

-----------------------------------------------------------------------------------------------
```

bmi

```
Body Mass Index
-----------------------------------------------------------------------------------------------

                  type:  numeric (float)

                 range:  [13.922607,50.561672]        units:  1.000e-06
         unique values:  891                      missing .:  0/898

                  mean:   23.0343
              std. dev:   5.03713

           percentiles:        10%       25%       50%       75%       90%
                           18.4381   19.7863   21.7339   24.9958   29.0832

-----------------------------------------------------------------------------------------------
```

```
bmicat4
Obese and others
-------------------------------------------------------------------------------------------------------

                type:  numeric (float)
               label:  bmicats1

               range:  [1,6]                       units:  1
       unique values:  4                       missing .:  0/898

          tabulation:  Freq.   Numeric  Label
                          96         1  <18.5 kg/m.sq.
                         578         2  18.5-24.99 kg/m.sq
                         150         3  25-29.99 kg/m.sq
                          74         6  >=30 kg/m.sq

-------------------------------------------------------------------------------------------------------
mn23sbp2
systolic blood pressure
-------------------------------------------------------------------------------------------------------

                type:  numeric (float)

               range:  [86,159]                    units:  1
       unique values:  59                      missing .:  0/898

                mean:   110.323
            std. dev:   10.1265

         percentiles:        10%       25%       50%       75%       90%
                              98       103       110       116       123

-------------------------------------------------------------------------------------------------------
mn23dbp2
diastolic bloos pressure
-------------------------------------------------------------------------------------------------------

                type:  numeric (float)

               range:  [27,125]                    units:  .1
       unique values:  61                      missing .:  0/898

                mean:   67.9488
            std. dev:   9.79945

         percentiles:        10%       25%       50%       75%       90%
                              56        61        69        74        80

-------------------------------------------------------------------------------------------------------

waistcirc2
waist circumference (cm)
-------------------------------------------------------------------------------------------------------

                type:  numeric (float)

               range:  [39.799999,167]             units:  1.000e-06
       unique values:  567                     missing .:  1/898

                mean:   74.4641
            std. dev:   11.5376

         percentiles:        10%       25%       50%       75%       90%
                         63.6333      67.1        72      78.8   88.5667

-------------------------------------------------------------------------------------------------------
```

```
hipcirc
hip circumference
--------------------------------------------------------------------------------------------------------------

                type:  numeric (float)

               range:  [30.799999,147.10001]        units:  1.000e-06
       unique values:  549                      missing .:  0/898

                mean:   95.4806
            std. dev:   10.1584

         percentiles:        10%       25%       50%       75%       90%
                            85.4      88.7   93.4667      99.8     109.2

--------------------------------------------------------------------------------------------------------------

whr
Waist-to-Hip Ratio
--------------------------------------------------------------------------------------------------------------
                type:  numeric (float)

               range:  [.27056423,3.6255412]        units:  1.000e-08
       unique values:  889                      missing .:  1/898

                mean:   .780465
            std. dev:   .117582

         percentiles:        10%       25%       50%       75%       90%
                         .713648   .742794    .77095   .806329      .841

--------------------------------------------------------------------------------------------------------------

inc_ldl
High LDL
--------------------------------------------------------------------------------------------------------------

                type:  numeric (float)
               label:  true

               range:  [0,1]                        units:  1
       unique values:  2                        missing .:  55/898

          tabulation:  Freq.   Numeric  Label
                         786         0  No
                          57         1  Yes
                          55         .

--------------------------------------------------------------------------------------------------------------

fastglu
fasting glucose
--------------------------------------------------------------------------------------------------------------
                type:  numeric (float)

               range:  [3.1,11.2]                   units:  .1
       unique values:  31                       missing .:  33/898

                mean:   4.57225
            std. dev:   .488353

         percentiles:        10%       25%       50%       75%       90%
                               4       4.3       4.5       4.8       5.1

--------------------------------------------------------------------------------------------------------------
```

fast_ins

```
fasting insulin
-------------------------------------------------------------------------------

                  type:  numeric (float)

                 range:  [2,79.4]                     units:  .1
         unique values:  164                      missing .:  115/898

                  mean:   6.93831
              std. dev:   5.87429

           percentiles:        10%       25%       50%       75%       90%
                                 2       3.3       5.8       8.8      12.6

-------------------------------------------------------------------------------
```

fast_chol

```
fasting cholesterol
-------------------------------------------------------------------------------

                  type:  numeric (float)

                 range:  [1.7,8.5]                    units:  .1
         unique values:  51                       missing .:  32/898

                  mean:    4.3343
              std. dev:   .872596

           percentiles:        10%       25%       50%       75%       90%
                               3.3       3.7       4.2       4.8       5.5

-------------------------------------------------------------------------------
```

fast_hdl

```
fasting HDL
-------------------------------------------------------------------------------

                  type:  numeric (float)

                 range:  [.42,2.5]                    units:  .01
         unique values:  125                      missing .:  50/898

                  mean:   1.20079
              std. dev:   .279558

           percentiles:        10%       25%       50%       75%       90%
                               .89      1.01      1.16      1.35      1.55

-------------------------------------------------------------------------------
```

fast_ldl

```
fasting LDL
-------------------------------------------------------------------------------

                  type:  numeric (float)

                 range:  [1.0111927,7.1616516]        units:  1.000e-07
         unique values:  833                      missing .:  55/898

                  mean:   2.87316
              std. dev:   .785512

           percentiles:        10%       25%       50%       75%       90%
                           1.97651   2.33642    2.7856   3.33101   3.91761

-------------------------------------------------------------------------------
```

```
fast_trig
fasting triglycerides
----------------------------------------------------------------------------------------------------------

                  type:  numeric (float)

                 range:  [.15,2.58]                   units:  .01
         unique values:  120                      missing .:  32/898

                  mean:   .581351
              std. dev:   .262237

           percentiles:        10%       25%       50%       75%       90%
                               .32        .4       .53        .7        .9

----------------------------------------------------------------------------------------------------------
```

```
htn
Hypertensive
----------------------------------------------------------------------------------------------------------

                  type:  numeric (float)
                 label:  true

                 range:  [0,1]                        units:  1
         unique values:  2                        missing .:  0/898

            tabulation:  Freq.   Numeric  Label
                           886         0  No
                            12         1  Yes

----------------------------------------------------------------------------------------------------------
```

```
smoke_current
currently smokes cigarettes
----------------------------------------------------------------------------------------------------------

                  type:  numeric (float)
                 label:  true

                 range:  [0,1]                        units:  1
         unique values:  2                        missing .:  1/898

            tabulation:  Freq.   Numeric  Label
                           811         0  No
                            86         1  Yes
                             1         .

----------------------------------------------------------------------------------------------------------
```

```
high_chol
High Total Cholesterol (>=5.2)
----------------------------------------------------------------------------------------------------------

                  type:  numeric (float)
                 label:  true

                 range:  [0,1]                        units:  1
         unique values:  2                        missing .:  21/898

            tabulation:  Freq.   Numeric  Label
                           751         0  No
                           126         1  Yes
                            21         .

----------------------------------------------------------------------------------------------------------
```

glucat_quin5
Upper Quintile of Fasting Glucose

```
---------------------------------------------------------------------------------------------------------

                 type:  numeric (float)
                label:  true

                range:  [0,1]                        units:  1
        unique values:  2                        missing .:  33/898

           tabulation:  Freq.   Numeric  Label
                          713         0  No
                          152         1  Yes
                           33         .

---------------------------------------------------------------------------------------------------------
```

chol_hdl_ratio
cholesterol to hdl ratio

```
---------------------------------------------------------------------------------------------------------
                 type:  numeric (float)

                range:  [1.7877096,10.365853]        units:  1.000e-07
        unique values:  656                      missing .:  55/898

                 mean:   3.76448
             std. dev:   1.02747

          percentiles:        10%       25%       50%       75%       90%
                          2.63889   3.10345   3.58381   4.31373   5.08333

---------------------------------------------------------------------------------------------------------
```

phys_act_level2
Physical Activity Level

```
---------------------------------------------------------------------------------------------------------

                 type:  numeric (float)
                label:  phys_act_level2

                range:  [1,3]                        units:  1
        unique values:  3                        missing .:  1/898

           tabulation:  Freq.   Numeric  Label
                          217         1  High PAL
                          374         2  Moderate PAL
                          306         3  Low PAL
                            1         .

---------------------------------------------------------------------------------------------------------
```

sex
sex

```
---------------------------------------------------------------------------------------------------------

                 type:  numeric (float)
                label:  sex

                range:  [0,1]                        units:  1
        unique values:  2                        missing .:  0/898

           tabulation:  Freq.   Numeric  Label
                          489         0  female
                          409         1  male

---------------------------------------------------------------------------------------------------------
```

alcoholcat

```
Alcohol Consumption Categories
-----------------------------------------------------------------------------------------------------------

                  type:  numeric (float)
                 label:  alcoholcat

                 range:  [0,3]                        units:  1
         unique values:  4                        missing .:  8/898

            tabulation:  Freq.   Numeric  Label
                            90         0  Never Drank Alcohol
                           326         1  Rarely Drinks Alcohol
                           226         2  Drinks Alcohol 1-2 times/week
                           248         3  Drinks Alcohol 3 or more
                                          times/week
                             8         .

-----------------------------------------------------------------------------------------------------------
```

trigcat_quin5

```
upper quintile of triglycerides
-----------------------------------------------------------------------------------------------------------

                  type:  numeric (float)
                 label:  true

                 range:  [0,1]                        units:  1
         unique values:  2                        missing .:  32/898

            tabulation:  Freq.   Numeric  Label
                           688         0  No
                           178         1  Yes
                            32         .

-----------------------------------------------------------------------------------------------------------
```

highbp_120_80

```
elevated blood pressure (>=120/80 mmHg)
-----------------------------------------------------------------------------------------------------------

                  type:  numeric (float)
                 label:  true

                 range:  [0,1]                        units:  1
         unique values:  2                        missing .:  0/898

            tabulation:  Freq.   Numeric  Label
                           710         0  No
                           188         1  Yes

-----------------------------------------------------------------------------------------------------------
```

famhx_htn

```
family history of hypertension
-----------------------------------------------------------------------------------------------------------
                  type:  numeric (float)
                 label:  true

                 range:  [0,1]                        units:  1
         unique values:  2                        missing .:  169/898

            tabulation:  Freq.   Numeric  Label
                           404         0  No
                           325         1  Yes
                           169         .

-----------------------------------------------------------------------------------------------------------
```

```
whtr
waist to height ratio
-----------------------------------------------------------------------------------------------
                type:  numeric (float)

               range:  [.2391827,.99345624]         units:  1.000e-08
       unique values:  895                      missing .:  1/898

                mean:   .439782
            std. dev:   .069724

         percentiles:        10%       25%       50%       75%       90%
                        .378234   .395082   .419896   .466123   .529521

-----------------------------------------------------------------------------------------------

wbc_count
white blood cell count
-----------------------------------------------------------------------------------------------

                type:  numeric (float)

               range:  [2,14.9]                     units:  .1
       unique values:  93                       missing .:  29/898

                mean:   5.87066
            std. dev:   1.87353

         percentiles:        10%       25%       50%       75%       90%
                             3.8       4.5       5.6       6.8       8.3

-----------------------------------------------------------------------------------------------
albumin_urine
urine albumin excretion (mg/g creat)
-----------------------------------------------------------------------------------------------

                type:  numeric (float)

               range:  [.6,816.9]                   units:  .1
       unique values:  237                      missing .:  38/898

                mean:   11.9241
            std. dev:   41.6842

         percentiles:        10%       25%       50%       75%       90%
                            1.85       2.5       4.1      9.15     22.55

-----------------------------------------------------------------------------------------------
micro_alb
Albuminuria
-----------------------------------------------------------------------------------------------

                type:  numeric (float)
               label:  micro_alb

               range:  [0,1]                        units:  1
       unique values:  2                        missing .:  38/898

          tabulation:  Freq.   Numeric  Label
                         801         0  No
                          59         1  Yes
                          38         .

-----------------------------------------------------------------------------------------------
```

gfr_lyon
Schwartz-Lyon

```
--------------------------------------------------------------------------------

                type:  numeric (float)

               range:  [10.467187,323.01001]        units:  1.000e-06
       unique values:  826                       missing .:  46/898

                mean:   94.9963
            std. dev:   29.0875

         percentiles:        10%       25%       50%       75%       90%
                         67.6059   76.1927   89.2246   105.429   126.121

--------------------------------------------------------------------------------
```

log_gfr_lyon
Log Schwartz-Lyon

```
--------------------------------------------------------------------------------

                type:  numeric (float)

               range:  [2.3482454,5.7776833]        units:  1.000e-07
       unique values:  826                       missing .:  46/898

                mean:   4.51529
            std. dev:   .271935

         percentiles:        10%       25%       50%       75%       90%
                          4.2137   4.33327   4.49116   4.65804   4.83724

--------------------------------------------------------------------------------
```

ckd
chronic kidney disease present

```
--------------------------------------------------------------------------------

                type:  numeric (float)
               label:  true

               range:  [0,1]                        units:  1
       unique values:  2                         missing .:  64/898

          tabulation:  Freq.   Numeric  Label
                         758         0  No
                          76         1  Yes
                          64         .

--------------------------------------------------------------------------------
```

ganja_current
currently smokes ganja

```
--------------------------------------------------------------------------------
                type:  numeric (float)
               label:  true

               range:  [0,1]                        units:  1
       unique values:  2                         missing .:  4/898

          tabulation:  Freq.   Numeric  Label
                         732         0  No
                         162         1  Yes
                           4         .

--------------------------------------------------------------------------------
```

```
binge
reports binge drinking of alcohol
-----------------------------------------------------------------------------------------------

                  type:  numeric (float)
                 label:  true

                 range:  [0,1]                        units:  1
         unique values:  2                        missing .:  102/898

            tabulation:  Freq.   Numeric  Label
                           585         0  No
                           211         1  Yes
                           102         .

-----------------------------------------------------------------------------------------------

hscrp_mn2
mean hscrp (values >10 set to missing)
-----------------------------------------------------------------------------------------------

                  type:  numeric (float)

                 range:  [.3,9.9]                     units:  .01
         unique values:  129                      missing .:  107/898

                  mean:    1.5012
              std. dev:   1.89852

           percentiles:        10%       25%       50%       75%       90%
                                .3        .3        .7       1.8      4.05

-----------------------------------------------------------------------------------------------


highest_ed_new
Highest Education Level of Parent/Guardian
-----------------------------------------------------------------------------------------------

                  type:  numeric (float)
                 label:  highest_ed2

                 range:  [1,3]                        units:  1
         unique values:  3                        missing .:  119/898

            tabulation:  Freq.   Numeric  Label
                           220         1  Tertiary
                           453         2  Secondary
                           106         3  Primary/All Age
                           119         .

-----------------------------------------------------------------------------------------------

occ_cat1a_new
occupation of household head
-----------------------------------------------------------------------------------------------
                  type:  numeric (float)
                 label:  occ_cat

                 range:  [1,3]                        units:  1
         unique values:  3                        missing .:  61/898

            tabulation:  Freq.   Numeric  Label
                           202         1  Highly Skilled
                           420         2  Skilled
                           215         3  Semi/Unskilled
                            61         .
-----------------------------------------------------------------------------------------------
```

possession_cat
possession score categories

```
------------------------------------------------------------------------------------------------------------

                type:  numeric (float)
               label:  possession_cat

               range:  [1,3]                        units:  1
       unique values:  3                        missing .:  1/898

          tabulation:  Freq.   Numeric  Label
                         136         1  15-17
                         497         2  10-14
                         264         3  0-9
                           1         .

------------------------------------------------------------------------------------------------------------
```

waist_glucose
central obesity + high glucose

```
------------------------------------------------------------------------------------------------------------

                type:  numeric (byte)
               label:  true

               range:  [0,1]                        units:  1
       unique values:  2                        missing .:  0/898

          tabulation:  Freq.   Numeric  Label
                         875         0  No
                          23         1  Yes

------------------------------------------------------------------------------------------------------------
```

waist_trig
central obesity + high triglycerides

```
------------------------------------------------------------------------------------------------------------

                type:  numeric (byte)
               label:  true

               range:  [0,1]                        units:  1
       unique values:  2                        missing .:  0/898

          tabulation:  Freq.   Numeric  Label
                         852         0  No
                          46         1  Yes

------------------------------------------------------------------------------------------------------------
```

waist_glucose_trig
central obesity + high glucose + high Trig

```
------------------------------------------------------------------------------------------------------------

                type:  numeric (byte)
               label:  true

               range:  [0,1]                        units:  1
       unique values:  2                        missing .:  0/898

          tabulation:  Freq.   Numeric  Label
                         887         0  No
                          11         1  Yes

------------------------------------------------------------------------------------------------------------
```

log_insulin

```
log fasting insulin
------------------------------------------------------------------------------------------------------------

                  type:  numeric (float)

                 range:  [.69314718,4.3744984]        units:  1.000e-08
         unique values:  164                      missing .:  115/898

                  mean:   1.70863
              std. dev:   .659837

           percentiles:        10%       25%       50%       75%       90%
                          .693147   1.19392   1.75786   2.17475    2.5337

------------------------------------------------------------------------------------------------------------
```

log_insulin_quin5

```
upper quintile of log insulin
------------------------------------------------------------------------------------------------------------

                  type:  numeric (byte)
                 label:  true

                 range:  [0,1]                        units:  1
         unique values:  2                        missing .:  0/898

            tabulation:  Freq.   Numeric  Label
                           741         0  No
                           157         1  Yes

------------------------------------------------------------------------------------------------------------
```

riskscore_highbp_new

```
number of risk factors (clustering) for high BP
------------------------------------------------------------------------------------------------------------

                  type:  numeric (float)
                 label:  riskscore_highbp3

                 range:  [0,3]                        units:  1
         unique values:  4                        missing .:  0/898

            tabulation:  Freq.   Numeric  Label
                           478         0  None
                           261         1  One
                           118         2  Two
                            41         3  Three or more

------------------------------------------------------------------------------------------------------------
```

waist_insulin

```
central obesity + high insulin
------------------------------------------------------------------------------------------------------------

                  type:  numeric (byte)
                 label:  true

                 range:  [0,1]                        units:  1
         unique values:  2                        missing .:  0/898

            tabulation:  Freq.   Numeric  Label
                           831         0  No
                            67         1  Yes

------------------------------------------------------------------------------------------------------------
```

```
waist_glucose_insulin
elevated waist glucose & insulin
------------------------------------------------------------------------------------------

                  type:  numeric (byte)
                 label:  true

                 range:  [0,1]                        units:  1
         unique values:  2                        missing .:  0/898

           tabulation:  Freq.   Numeric  Label
                          886         0  No
                           12         1  Yes

------------------------------------------------------------------------------------------

waist_trig_insulin
elevated waist triglyceride & insulin
------------------------------------------------------------------------------------------

                  type:  numeric (byte)
                 label:  true

                 range:  [0,1]                        units:  1
         unique values:  2                        missing .:  0/898

           tabulation:  Freq.   Numeric  Label
                          875         0  No
                           23         1  Yes

------------------------------------------------------------------------------------------

waist_glucose_trig_insulin
central obesity + high glucose + high trig + high insulin
------------------------------------------------------------------------------------------

                  type:  numeric (byte)
                 label:  true

                 range:  [0,1]                        units:  1
         unique values:  2                        missing .:  0/898

           tabulation:  Freq.   Numeric  Label
                          891         0  No
                            7         1  Yes

------------------------------------------------------------------------------------------

high_hscrp2
high hsCRP (values >10 excluded)
------------------------------------------------------------------------------------------
type:  numeric (float)
                 label:  true

                 range:  [0,1]                        units:  1
         unique values:  2                        missing .:  107/898

           tabulation:  Freq.   Numeric  Label
                          677         0  No
                          114         1  Yes
                          107         .

------------------------------------------------------------------------------------------
```
